# Seroprevalence of Antibodies to SARS-CoV-2 in Rural Households in Eastern Uganda, 2020-2022

**DOI:** 10.1001/jamanetworkopen.2022.55978

**Published:** 2023-02-15

**Authors:** Jessica Briggs, Saki Takahashi, Patience Nayebare, Gloria Cuu, John Rek, Maato Zedi, Timothy Kizza, Emmanuel Arinaitwe, Joaniter I. Nankabirwa, Moses Kamya, Prasanna Jagannathan, Karen Jacobson, Philip J. Rosenthal, Grant Dorsey, Bryan Greenhouse, Isaac Ssewanyana, Isabel Rodríguez-Barraquer

**Affiliations:** 1 Division of HIV, ID, and Global Medicine, Department of Medicine, University of California San Francisco, San Francisco; 2 Infectious Diseases Research Collaboration, Kampala, Uganda; 3 Department of Medicine, Makerere University, Kampala, Uganda; 4 Department of Medicine, Stanford University, Stanford, California; 5 Department of Microbiology and Immunology, Stanford University, Stanford, California; 6 Central Public Health Laboratories, Butabika, Uganda

## Abstract

**Importance:**

Estimating the true burden of SARS-CoV-2 infection has been difficult in sub-Saharan Africa owing to asymptomatic infections and inadequate testing capacity. Antibody responses from serologic surveys can provide an estimate of SARS-CoV-2 exposure at the population level.

**Objective:**

To estimate SARS-CoV-2 seroprevalence, attack rates, and reinfection in eastern Uganda using serologic surveillance from 2020 to early 2022.

**Design, Setting, and Participants:**

This cohort study was conducted in the Tororo and Busia districts of eastern Uganda. Plasma samples from participants in the Program for Resistance, Immunology, Surveillance, and Modeling of Malaria in Uganda Border Cohort were obtained at 4 sampling intervals: October to November 2020, March to April 2021, August to September 2021, and February to March 2022. Each participant contributed up to 4 time points for SARS-CoV-2 serology, with almost half of all participants contributing at all 4 time points, and almost 90% contributing at 3 or 4 time points. Information on SARS-CoV-2 vaccination status was collected from participants, with the earliest reported vaccinations in the cohort occurring in May 2021.

**Main Outcomes and Measures:**

The main outcomes of this study were antibody responses to the SARS-CoV-2 spike protein as measured with a bead-based serologic assay. Individual-level outcomes were aggregated to population-level SARS-CoV-2 seroprevalence, attack rates, and boosting rates. Estimates were weighted by the local age distribution according to census data.

**Results:**

A total of 1483 samples from 441 participants living in 76 households were tested. Of the 441 participants, 245 (55.6%) were female, and their mean (SD) age was 16.04 (16.04) years. By the end of the Delta wave and before widespread vaccination, adjusted SARS-CoV-2 seroprevalence was 67.7% (95% credible interval [CrI], 62.5%-72.6%) in the study population. During the subsequent Omicron wave, 84.8% (95% CrI, 67.9%-93.7%) of unvaccinated, previously seronegative individuals were infected for the first time, and 50.8% (95% CrI, 40.6%-59.7%) of unvaccinated, already seropositive individuals were likely reinfected, leading to an overall seropositivity of 96.0% (95% CrI, 93.4%-97.9%) in this population. These results suggest a lower probability of reinfection in individuals with higher preexisting antibody levels. There was evidence of household clustering of SARS-CoV-2 seroconversion. No significant associations were found between SARS-CoV-2 seroconversion and gender, household size, or recent *Plasmodium falciparum* malaria exposure.

**Conclusions and Relevance:**

In this cohort study in a rural population in eastern Uganda, there was evidence of very high SARS-CoV-2 infection rates throughout the pandemic inconsistent with national level case data and high reinfection rates during the Omicron wave.

## Key Points

**Question:**

What proportion of a rural population in eastern Uganda has been infected and reinfected with SARS-CoV-2?

**Findings:**

In this cohort study of 441 participants, by the end of the SARS-CoV-2 Delta wave and before the widespread availability of vaccination, 67.7% of the cohort study population had been infected with SARS-CoV-2. During the subsequent Omicron wave in early 2022, 84.8% of unvaccinated, previously seronegative individuals were infected for the first time, and 50.8% of unvaccinated, already seropositive individuals were likely reinfected.

**Meaning:**

These results contribute to a growing body of seroprevalence data from Africa suggesting that there are very high SARS-CoV-2 infection rates despite low case ascertainment.

## Teaser

This cohort study estimates SARS-CoV-2 seroprevalence, attack rates, and reinfection in rural eastern Uganda using serologic surveillance from 2020 to early 2022.

## Introduction

Understanding population-level exposure and immunity to SARS-CoV-2 is necessary to measure transmission, determine the susceptible population, and inform public health responses. However, estimating the proportion of the population that has been infected, particularly in resource-limited settings, is complicated by asymptomatic or subclinical infections, inadequate testing capacity, and challenges in collecting routine surveillance data. Seroprevalence surveys can overcome these issues by identifying antibody responses that reflect prior SARS-CoV-2 exposure.^1^ A recent review of seroprevalence studies conducted in sub-Saharan Africa when vaccination coverage was low (<5% in September 2021) reported an increase in SARS-CoV-2 seroprevalence from 3% in the second quarter of 2020 to 65% in the third quarter of 2021.^2^

There are few reported SARS-CoV-2 seroprevalence data specific to Uganda to elucidate the true burden of infection and underascertainment of cases. Only 1 population-level serosurvey in Uganda has been published thus far, which identified an overall seroprevalence of 21% in March 2021.^3^ To our knowledge, there have also been no published serosurveys in sub-Saharan Africa that estimate population seroprevalence after the Omicron wave or the attack rate of the Omicron variant. Here, we leveraged samples collected as part of a longitudinal malaria cohort study in eastern Uganda to reconstruct the epidemic trajectory of SARS-CoV-2 over the first 2 years of the pandemic and estimate the attack rate of the main epidemic waves, along with risk factors for seroconversion.

## Methods

### Study Design

The study protocol was approved by the Makerere University School of Medicine Research and Ethics Committee, the Uganda National Council of Science and Technology, the University of California, San Francisco, Human Research Protection Program, the London School of Hygiene & Tropical Medicine Ethics Committee, and the Stanford University Research Compliance Office. Written informed consent was obtained for all participants before study enrollment. This study followed the Strengthening the Reporting of Observational Studies in Epidemiology (STROBE) reporting guideline for cohort studies.

This cohort study was conducted within the Program for Resistance, Immunology, Surveillance, and Modeling of Malaria in Uganda (PRISM) Border Cohort study in the Tororo and Busia districts of Uganda. The design and population of this ongoing cohort have recently been described elsewhere.^4^ At cohort study enrollment, a survey was conducted to collect information on household characteristics, including household wealth tertile,^5,6^ sanitation category, and housing type (traditional vs modern).

Participants from the 80 households enrolled in the cohort were encouraged to come to a dedicated study clinic open 7 days per week for all medical care, which they received free of charge. Routine study visits were conducted every 4 weeks and included a standardized evaluation and blood collection. Information on SARS-CoV-2 vaccination status and dates was collected from participants.

**Figure 1.**
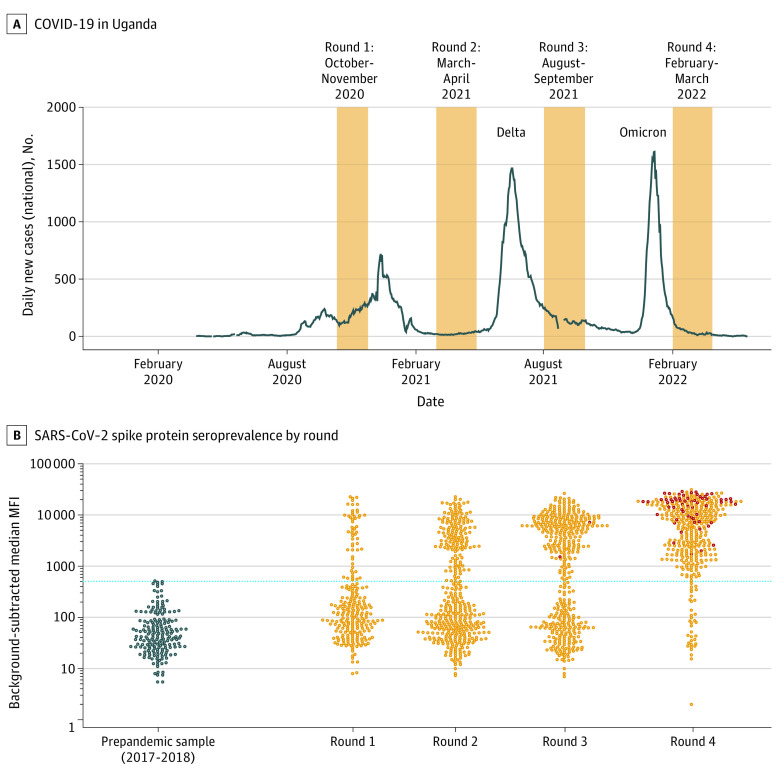
The SARS-CoV-2 Epidemic in Uganda and in the Program for Resistance, Immunology, Surveillance, and Modeling of Malaria (PRISM) Border Cohort Study A, Reported daily new COVID-19 cases in Uganda (7-day rolling average), obtained from the Our World in Data database.^7^ One week in late August 2021 when more than 21 000 cases were reported in 1 day is omitted. The 4 rounds of the PRISM Border Cohort SARS-CoV-2 serosurveys are shaded orange. B, SARS-CoV-2 seroprevalence by round. The y-axis shows the background-subtracted median mean fluorescent intensity (MFI) of the spike protein antibody response. The left-most bee-swarm plot (dark blue circles) shows responses from 192 prepandemic control samples from the PRISM-2 study (also in Tororo District, Uganda), collected in 2017 to 2018. The subsequent bee-swarm plots show the distribution of antibody responses by serosurvey round. Visits from participants who had received SARS-CoV-2 vaccination by 3 weeks before the serosurvey sample (ie, to allow for seroconversion after vaccination) are shown as dark red circles. The cutoff for seropositivity is shown in the blue dotted line (background-subtracted median MFI = 516). The raw seroprevalence was 22.0% in round 1, 37.9% in round 2, 59.9% in round 3, and 91.0% in round 4.

For this SARS-CoV-2 study, we leveraged the biospecimen repository of plasma samples collected at monthly routine study visits. We selected 4 sampling intervals between October 2020 and March 2022, informed by COVID-19 case counts in Uganda, obtained from the Our World in Data database^7^ (Figure 1A and eFigure 1 in Supplement 1). For sampling, we included all available samples from study participants who had a routine visit during the round 3 sampling interval.

### Laboratory Methods

A multiplex bead assay and protocol previously described for SARS-CoV-2 serology studies^8,9^ was modified to assess total IgG responses to the spike protein, the receptor-binding domain of the spike protein, and the nucleocapsid protein. Detailed methods are in the eAppendix in Supplement 1. Briefly, plasma samples were assayed at a 1:400 dilution to determine antibody seropositivity. The results were expressed as mean fluorescent intensity. A standard curve using a pool of positive serum was included on each plate to normalize for plate-to-plate variations (eFigure 2 in Supplement 1) and to infer relative antibody concentrations using a 4-parameter logistic model.^10^ Samples run in duplicate demonstrated high replicability (eFigure 3 in Supplement 1). A subset of samples was tested at a 1:4000 dilution to assess antibody boosting. A greater dilution for the boosting assay was used, because many seropositive samples were near or outside the assay’s linear dynamic range at 1:400.

For negative controls, we used 192 prepandemic plasma samples from the PRISM-2 cohort study, also in Tororo District, Uganda,^11^ collected in 2017 and 2018 (eFigure 4 in Supplement 1). These samples were selected to have the same age distribution as the study population and represented an appropriate background for serologic signals (eg, cross-reactivity from other circulating coronaviruses).^12^ For positive controls, we used 156 plasma samples from 99 volunteers enrolled in the UCSF Long-term Impact of Infection with Novel Coronavirus study.^8^ Cutoff for seropositivity was established as the highest value among the negative controls, and, therefore, specificity was 100% (192 of 192 controls) by design. Assay sensitivity was calculated as the proportion of the positive controls above the cutoff, and was 92.9% (145 of 156 controls) (eFigure 5 in Supplement 1). As an additional check on assay performance characteristics, we calculated the sensitivity in samples from 11 participants from the PRISM Border Cohort study who tested positive for symptomatic SARS-CoV-2 infection between February and July 2021.^13^ Ten of 11 participants (90.9%) seroconverted, and responses remained robust over time (eFigure 6 in Supplement 1).

### Statistical Analysis

We focused on spike antibody responses and used background-subtracted mean fluorescent intensities as the primary outcome. A sensitivity analysis was performed using relative concentrations as the outcome and demonstrated highly concordant results (eFigure 7 in Supplement 1). Although the assay also measured receptor-binding domain and nucleocapsid responses, the receptor-binding domain and spike responses were highly correlated, and the nucleocapsid responses had high background in this assay, as previously noted,^8^ and, thus, were not well suited to evaluate primary seroconversion to natural infection (eFigure 8 in Supplement 1).

Raw SARS-CoV-2 spike seropositivity was determined as the proportion of samples that tested positive at the 1:400 dilution. We then estimated seroprevalence, adjusted for sensitivity and specificity, using a bayesian measurement model.^14^ We also used this model to estimate the attack rate, defined as the proportion of individuals seronegative in 1 serosurvey round who were seropositive at the subsequent round. We computed 95% credible intervals (CrIs) to quantify uncertainty in posterior estimates. We produced estimates weighted by the local age distribution on the basis of data from the most recent census in 2014 from the Uganda Bureau of Statistics.^15^

To assess SARS-CoV-2 boosting, we analyzed responses at the 1:4000 dilution. We limited assessment of boosting to between rounds 3 and 4 in individuals who were already seropositive at round 3. In the primary analysis, we defined boosting as a 4-fold or higher increase in response, a commonly used threshold across pathogens for ascertaining recent infection.^16,17,18^ In sensitivity analyses, we assessed alternative definitions (ie, ≥2-fold and ≥8-fold increases) and incorporating nucleocapsid responses. Because participants were receiving vaccines during this interval, nucleocapsid responses were included in evaluation of boosting (but not primary seroconversion) because they can potentially differentiate antibody responses resulting from infection vs vaccination with spike-based vaccines. The latter do not generate nucleocapsid responses and include the majority of SARS-CoV-2 vaccines received by participants in this cohort.

In analyses to test for individual and household characteristics (and visit-level patient-reported symptoms and clinician-assigned diagnoses) associated with SARS-CoV-2 seroconversion and boosting, univariate binomial regression was performed. For associations with seroconversion, patients could contribute observations from multiple rounds until seroconversion; therefore, generalized estimating equations were used to adjust for repeated measures.^19^ For associations with boosting, only children were included because SARS-CoV-2 vaccination had begun in those aged 18 years and older by the time of sample collection. Malaria incidence between each sampling round was calculated by dividing the number of episodes of clinical malaria by the number of days of observation for each individual. Asymptomatic parasitemia was defined as the presence of *Plasmodium falciparum* parasites via microscopy or *var* gene acidic terminal sequence quantitative polymerase chain reaction^20^ and the absence of fever (measured or by history).

To test for clustering of seroconversions within households, we adapted an approach of estimating pairwise odds ratios (ORs) to detect correlation structure in binary data^21,22,23^ (see eAppendix in Supplement 1). Two-sided *P* < .05 was considered significant. All analyses were conducted using R statistical software version 4.2.0 (R Project for Statistical Computing)^24^ and the Stan programming language version 2.21.2 (The Stan Development Team).^25^

## Results

### Clinical and Demographic Characteristics of the Study Participants

A total of 1483 samples from 441 participants (245 female [55.6%]; mean [SD] age, 16.04 [16.04] years) living in 76 households were tested (Table 1). Participants contributed up to 4 time points (Figure 1A), with almost half of participants contributing at all 4 time points, and almost 90% contributing at 3 or 4 time points. Household sizes ranged from 3 to 8 residents. Results had approximately equal representation from those younger than 5 years, those aged 5 to 15 years, and those aged 16 years or older at the time of their first sample collection. The earliest reported SARS-CoV-2 vaccinations in the cohort occurred in May 2021: 3 adults were vaccinated between round 2 (March-April 2021) and round 3 (August-September 2021), and 105 of 137 adults (77%) included in round 4 (February-March 2022) received SARS-CoV-2 vaccination between rounds 3 and 4 (eFigure 9 in Supplement 1). One participant younger than 15 years received SARS-CoV-2 vaccination at 2 weeks before their round 4 sample. The distribution of manufacturers of vaccines received were 83 Janssen/Johnson & Johnson, 12 AstraZeneca, 9 Moderna, and 1 Sinovac. Although SARS-CoV-2 diagnostic testing was not systematically performed, 7 participants reported having tested positive by rapid diagnostic test. There were no hospitalizations or deaths due to COVID-19 in the cohort.

**Table 1.** Demographic Characteristics of the Study Participants

Characteristic	Participants, No. (%) (N = 441)
Age at first time point included, y	
<5	156 (35.4)
5-15	130 (29.5)
≥16	155 (35.1)
Sex	
Female	245 (55.6)
Male	196 (44.4)
District	
Tororo	350 (79.4)
Busia	91 (20.6)
Participants in a household, No. (n = 76 households)	
3	2 (2.6)
4	12 (15.8)
5	16 (21.1)
6	17 (22.4)
7	27 (35.5)
8	2 (2.6)
Serosurvey rounds, No. of samples	
Total	1483
Round 1 (October 12 to November 25, 2020)	245
Round 2 (March 2 to April 28, 2021)	414
Round 3 (August 2 to September 29, 2021)	434
Round 4 (February 1 to March 29, 2022)	390
Time points included per participant, No.	
1	4 (0.9)
2	46 (10.4)
3	177 (40.1)
4	214 (48.5)

### SARS-CoV-2 Seroprevalence, Attack Rates, and Boosting

**Figure 2.**
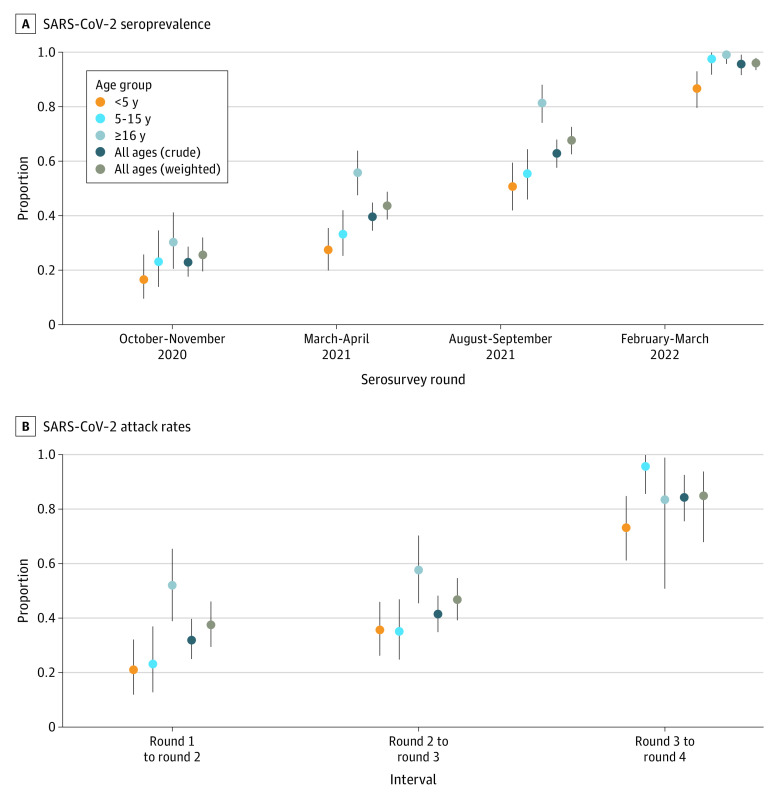
SARS-CoV-2 Seroprevalence and Attack Rates by Age Group and Serosurvey Round A, Graph shows posterior median (dots) and 95% credible intervals (error bars) for seroprevalence based on spike protein mean fluorescent intensity (MFI), accounting for test performance characteristics. B, Graph shows posterior median (dots) and 95% credible intervals (error bars) for attack rate based on spike protein MFIs. Individuals who had received SARS-CoV-2 vaccination by a serosurvey round are removed from the attack rate estimation (eg, individuals who were vaccinated at round 4 are removed from the attack rate estimation between round 3 and round 4). The colors represent age group-specific estimates. The dark blue values represent the crude estimates in the cohort. The brown values represent estimates weighted by the local age distribution using 2014 census data from the 3 parishes in Uganda in which study participants reside. All estimates are adjusted for test performance characteristics.

The raw seropositivity to spike was 22.0% during round 1 (October-November 2020, half a year since the first reported case in Uganda) and increased over time, reaching 91.0% seropositivity during round 4 (after the first Omicron wave) (Figure 1B and eTable 1 in Supplement 1). Weighting by the age distribution of the population and accounting for test performance characteristics, seroprevalence increased from 25.6% (95% CrI, 19.6%-32.0%) at round 1, to 43.7% (95% CrI, 38.6%-48.8%) at round 2, to 67.7% (95% CrI, 62.5%-72.6%) at round 3, and to 96.0% (95% CrI, 93.4%-97.9%) at round 4 (Figure 2A). The weighted seroprevalence among unvaccinated individuals was 68.2% (95% CrI, 62.7%-73.4%%) in round 3 and 94.7% (95% CrI, 88.3%-97.7%) in round 4. A total of 163 878 SARS-CoV-2 cases were reported in Uganda through March 29, 2022. Assuming that our estimated weighted seroprevalence in unvaccinated individuals holds at the national level, we estimate that only 1 in 270 infections were ascertained by the reporting system.

We used longitudinal antibody responses to identify seroconversions (eFigure 10 in Supplement 1) and to estimate the population-level attack rate of the main epidemic waves (Figure 2B and eTable 2 in Supplement 1). The age-weighted attack rate of the Delta wave (round 2 to round 3) was estimated to be 46.7% (95% CrI, 39.1%-54.6%). The age-weighted attack rate of the Omicron wave (round 3 to round 4), omitting individuals who were vaccinated at round 4, was estimated to be 84.8% (95% CrI, 67.9%-93.7%).

Among the 232 participants who were seropositive at round 3 and had a round 4 sample, we looked for evidence of antibody boosting between these rounds. A total of 125 of 232 participants (53.9%) were boosted on the basis of our primary definition of 4-fold or higher increase in mean fluorescent intensity (eTable 3 in Supplement 1). This included 70 of 84 participants vaccinated by round 4 and 55 of 148 participants who had not been vaccinated, consistent with reinfection. Excluding vaccine recipients, and weighting by the age distribution of the population, we estimated that at least 50.8% (95% CrI, 40.6%-59.7%) of individuals may have been reinfected during the Omicron wave.

**Figure 3.**
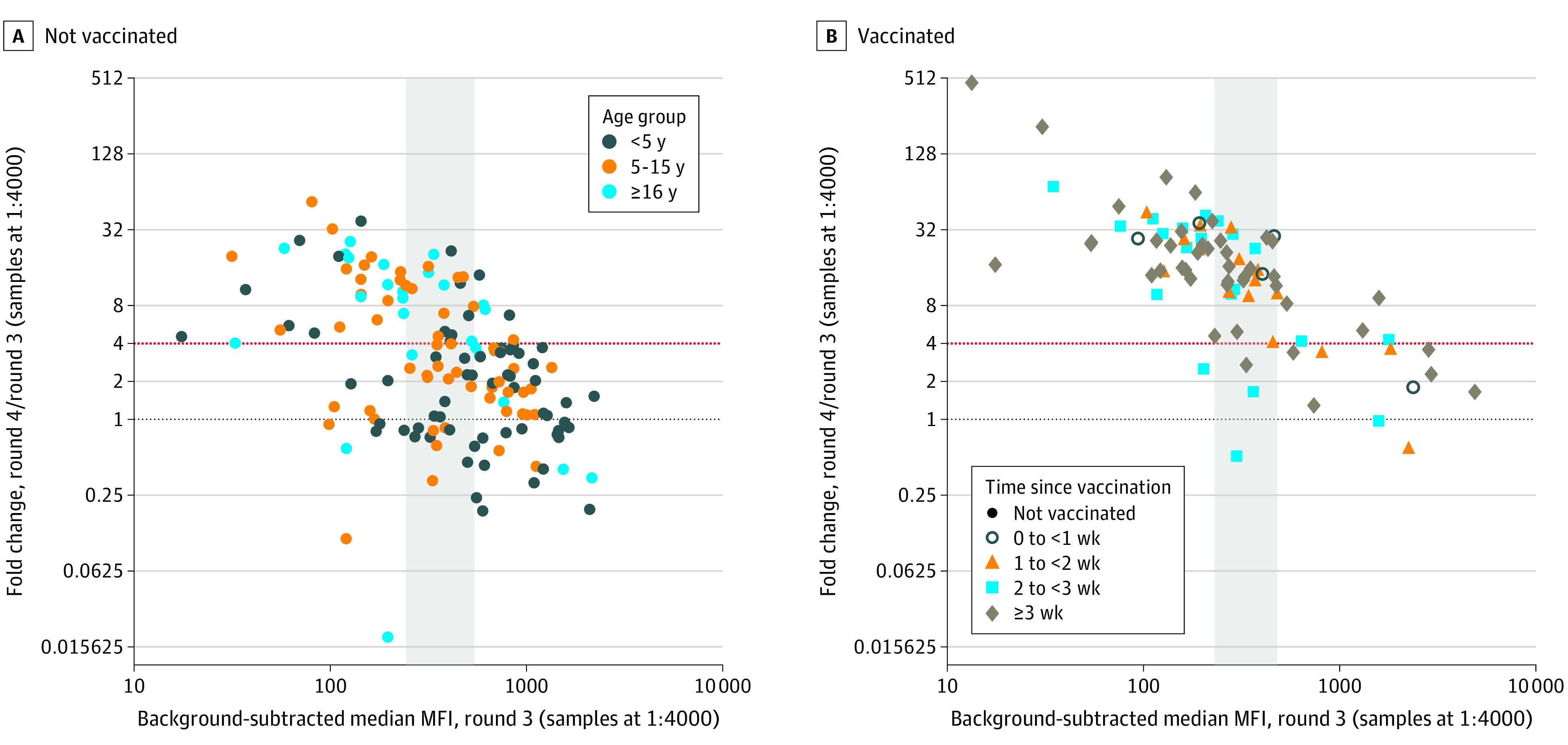
SARS-CoV-2 Antibody Boosting Between Rounds 3 and 4 A, Round 4 antibody responses among the 232 participants who were seropositive at round 3 (65 participants aged <5 years, 58 participants aged 5-15 years, and 109 participants aged ≥16 years) are shown. The round 3 spike protein antibody response is shown on the x-axis, and the fold change between the round 4 and round 3 spike antibody responses is shown on the y-axis. We defined boosting as a 4-fold or greater increase (red dotted line). B, Participants were separated by vaccination status at round 4 and by tertiles of round 3 response (the second tertile is shown in the gray shaded rectangle). The colors of the points represent age groups, and the shapes of the points represent binned time since SARS-CoV-2 vaccination at the round 4 sample. Note that in panel B, boosting was observed in 41 of 47 participants vaccinated more than 3 weeks before their round 4 sample was collected. Among individuals who were not vaccinated, the proportion who demonstrated antibody boosting was 71% in the first tertile, 42% in the second tertile, and 11% in the third tertile. Among vaccinated individuals, the proportion who demonstrated antibody boosting was 97% in the first tertile, 91% in the second tertile, and 33% in the third tertile.

Interestingly, we observed more boosting events among individuals who had lower antibody responses at round 3 than among individuals with higher responses (Figure 3 and eFigure 11 in Supplement 1). This was consistent across vaccination status, but was particularly evident among unvaccinated individuals. Although boosting was observed among 29 of 41 unvaccinated individuals (71%) with antibody levels in the lowest tertile, it was only observed in 7 of 62 unvaccinated individuals (11%) with antibody levels in the highest tertile. In addition to tertile, boosting risk increased by age (eFigure 12 in Supplement 1). Alternate estimates of boosting using different thresholds and incorporating nucleocapsid responses are shown in eFigures 13 and 14 in Supplement 1.

### Individual and Household Level Risk Factors for SARS-CoV-2 Seroconversion and Boosting

We found evidence of clustering of SARS-CoV-2 seroconversions within households (eFigure 15 in Supplement 1). Pooled across time intervals and age groups, the odds of seroconversion were 4.33 (95% CrI, 3.27-5.79) times greater in individuals from households where another individual seroconverted during the same period, compared with households where other individuals did not seroconvert. Clustering was strongest between child-child pairs (OR, 6.42; 95% CrI, 4.31-9.65), followed by child-adult pairs (OR, 2.92; 95% CrI, 1.85-4.64), and was lowest in adult-adult pairs (OR, 2.45; 95% CrI, 0.77-8.06), implying that 2 children in a household were more likely to seroconvert in the same interval than 2 adults. These findings suggest an elevated within-household secondary attack rate, particularly among children.^26^

In univariate analyses, compared with children younger than 5 years, older age was associated with SARS-CoV-2 seroconversion (OR, 1.77 [95% CI, 1.15-2.74] for adults aged ≥16 years; OR, 1.31 [95% CI, 0.89-1.94] for children aged 5-15 years) (Table 2). At each round, seroprevalence was highest among adults, followed by older children, and lowest in younger children (Figure 2A). Attack rates were considerably higher in adults during the first 2 intervals, but not the final interval (Figure 2B). We did not find an association between gender and seroconversion. No household characteristics evaluated were associated with seroconversion (Table 2).

**Table 2.** Individual and Household Level Risk Factors for SARS-CoV-2 Seroconversion

Characteristic	OR (95% CI)^a^	*P* value
Age category, y		
<5	1 [Reference]	NA
5-15	1.31 (0.89-1.94)	.17
≥16	1.77 (1.15-2.74)	.01
Sex		
Female	1 [Reference]	.70
Male	0.94 (0.67-1.31)	
Wealth tertile		
Lowest	1 [Reference]	NA
Middle	0.90 (0.60-1.35)	.62
Highest	0.90 (0.59-1.36)	.60
Housing type		
Modern	1 [Reference]	.11
Traditional	0.75 (0.53-1.07)	
Sanitation		
Uncovered pit latrine or no facility	1 [Reference]	.15
Ventilated improved pit or covered pit latrine	1.34 (0.90-2.00)	
No. of people in household	0.98 (0.86-1.13)	.82
Malaria incidence	0.99 (0.90-1.08)	.83
Any asymptomatic parasitemia	1.03 (0.73-1.45)	.88
No. of episodes of asymptomatic parasitemia	1.03 (0.89-1.19)	.71

Abbreviations: NA, not applicable; OR, odds ratio.

^a^ Generated from univariate binomial regression with generalized estimating equations to adjust for repeated measures.

To assess potential associations between SARS-CoV-2 infection and recent *P falciparum* infection, we examined the association between recent symptomatic malaria and asymptomatic parasitemia with SARS-CoV-2 seroconversion. Malaria incidence and number of episodes of asymptomatic parasitemia were calculated for each SARS-CoV-2–seronegative individual within each period between sampling rounds. There was no association between an individual’s incidence of symptomatic malaria or occurrence of asymptomatic parasitemia between sampling round and SARS-CoV-2 seroconversion (Table 2).

We explored associations between self-reported symptoms and clinician-assigned diagnoses with SARS-CoV-2 seroconversion during a time interval. Symptoms associated with SARS-CoV-2 seroconversion included cough, headache, and fatigue (eTable 4 in Supplement 1). The number of sick visits between sampling rounds, any upper respiratory tract infection diagnosis, and total number of upper respiratory tract infections were also associated with seroconversion.

Finally, these individual and household characteristics were investigated for association with boosting of the SARS-CoV-2 antibody response between rounds 3 and 4 in children (eTable 5 in Supplement 1); findings were similar to those in the seroconversion analysis. However, there was an association between antibody boosting and total number of asymptomatic malaria episodes in the period between round 3 and round 4 sampling (OR, 1.37; 95% CI, 1.05-1.81); this association remained but was attenuated after adjusting for age (OR, 1.29; 95% CI, 0.97-1.73). There were no specific symptoms or diagnoses associated with increased odds of antibody boosting.

## Discussion

Findings from this cohort study are consistent with very high attack rates of SARS-CoV-2 infection in this rural population from eastern Uganda throughout the pandemic. By the end of the SARS-CoV-2 Delta wave and before the widespread availability of vaccination, 67.7% of the study population had experienced SARS-CoV-2 infection. Moreover, during the subsequent Omicron wave in early 2022, 84.8% of unvaccinated, previously seronegative individuals were infected for the first time, and 50.8% of unvaccinated, already seropositive individuals were likely reinfected. On the basis of the SARS-CoV-2 cases reported in Uganda during the time period of this study (163 878 through March 29, 2022) and assuming our estimated weighted seroprevalence in unvaccinated individuals (94.7% at the end of round 4) holds at the national level, we estimate that only about 1 in 270 infections were ascertained by the reporting system.

The availability of longitudinal sampling enabled us to assess seroconversions and reinfections over time. Interestingly, our results based on serologic evidence of boosting suggested a lower probability of reinfection in individuals with higher preexisting antibody levels; similar patterns have previously been described in a triple-vaccinated population.^27^ Our observation could be explained by several potential factors, including immunity (ie, more effective viral neutralization among individuals with higher preexisting antibody titers), infection histories with different variants that may affect subsequent boosting,^28^ antibody homeostasis (ie, a ceiling for antibody titers), or limited dynamic range of the assay. Of these, we can only rule out the last explanation, because antibody responses in individuals who did not boost were far from the top of the dynamic range of this assay. If immunity is indeed a factor associated with this observation, it underscores the importance of achieving and maintaining sufficiently high antibody titers in the population (ie, through vaccination and revaccination at an appropriate frequency).

### Limitations

Our study has some important limitations. First, the samples were collected from 76 households in eastern Uganda, and, therefore, generalizability may be limited. In addition, the fact that we only tested samples obtained at approximately 5-month intervals and, therefore, do not know the exact timing of SARS-CoV-2 infection, limits our capacity to make precise inferences about associations between SARS-CoV-2 infection and clinical presentation. Similarly, although a protective association between malaria and COVID-19 has recently been hypothesized,^29,30,31^ in this study we could not directly test whether *P falciparum* infection status is associated with the risk of SARS-CoV-2 infection or disease. However, we were able to examine whether recent *P falciparum* infection history was associated with SARS-CoV-2 seroconversion. We found no definitive evidence of an association between recent *P falciparum* infection history and SARS-CoV-2 infection, despite an association between asymptomatic parasitemia and boosting during the Omicron wave in individuals younger than 16 years. Understanding potential immunologic interactions between these pathogens remains an important area of inquiry,^29,30^ particularly in this setting where exposures to the malaria parasite are high.^4^

Our results suggest a higher attack rate during the first Omicron wave of SARS-CoV-2 than the Delta wave (84.8% vs 46.7%), and the high estimated reinfection rate during Omicron (50.8%) is consistent with the evidence of increased infectivity and immune evasion associated with this variant.^32,33^ Because of the antigenic changes in the spike protein of the Omicron variant, the sensitivity of existing assays measuring spike antibody responses may be reduced against this variant.^34^ Reference standard serologic assays are needed to accurately determine reinfections, possibly with variant-specific assays. This will be increasingly important as reinfections become more frequent and new variants that potentially evade preexisting immunity continue to emerge. We did not have data to adjust for potential variant-specific reduced sensitivity or to incorporate seroreversions (although here and in previous work,^8^ we found this assay to demonstrate stable responses over the first few months following infection). Either would lead to increased estimates of seroprevalence, attack rates, and boosting.

## Conclusions

The results of this cohort study contribute to a growing body of seroprevalence data from low-resource settings that illustrate very high SARS-CoV-2 infection rates despite low case ascertainment. Infection histories have ultimately generated complex landscapes of SARS-CoV-2 hybrid immunity in different populations. It will be increasingly important to consider additional complexities that have since become central for interpreting the results of SARS-CoV-2 serosurveys, including histories of vaccination, breakthrough infection, and reinfection.

## Notes

Supplement 1. **eAppendix.** Supplemental Methods **eTable 1.** Number of Samples Seropositive for SARS-CoV-2 by Spike Protein MFI and Total Number of Samples Tested, by Serosurvey Round and Age Group **eTable 2.** Number of Participants With SARS-CoV-2 Seroconversion by Spike MFI and Total Number of Participants at Risk for Seroconversion, by Interval and Age Group **eTable 3.** Number of Participants With SARS-CoV-2 Boosting by Spike MFI and Total Number of Participants at Risk for Boosting, by Interval and Age Group **eTable 4.** Symptoms and Diagnoses Associated With SARS-CoV-2 Seroconversion **eTable 5.** Risk Factors for Antibody Boosting and Associations With Malaria **eFigure 1.** District-Level Weekly SARS-CoV-2 Case Counts in Eastern Uganda, Compared to in the Country Overall **eFigure 2.** Standard Dilution Series Used for Assay Normalization **eFigure 3.** Replicability of the Luminex Assay **eFigure 4. **The Age Distribution of This Study Population, Compared to the Age Distribution of the Negative Control Samples Used **eFigure 5.** Spike Protein Antibody Responses for SARS-CoV-2 Negative Control and Positive Control Samples **eFigure 6.** Longitudinal Antibody Kinetics for 11 Participants in the Cohort Study Who Had Confirmed SARS-CoV-2 Infections **eFigure 7.** Scatterplot of Agreement Between Measured MFIs and Inferred Antibody Concentrations **eFigure 8.** Antibody Responses to Additional SARS-CoV-2 Antigens Tested **eFigure 9.** Timing of SARS-CoV-2 Vaccination **eFigure 10.** SARS-CoV-2 Antibody Kinetics, Stratified by Age Group and by Serosurvey Round at Which an Individual’s Antibody Response Was First Positive **eFigure 11.** SARS-CoV-2 Antibody Boosting Between Rounds 3 and 4 in Vaccinated Individuals, Stratified by Vaccine Type **eFigure 12.** Antibody Boosting by Strata **eFigure 13.** Sensitivity Analyses for SARS-CoV-2 Antibody Boosting Between Rounds 3 and 4 Using Alternative Cutoffs **eFigure 14.** Sensitivity Analyses for SARS-CoV-2 Antibody Boosting Between Rounds 3 and 4 Using Nucleocapsid Protein Responses **eFigure 15. **Pairwise Odds Ratios for Clustering of SARS-CoV-2 Seroconversions Within Households, by Time Period and by Age Category

Supplement 2.
Data Sharing Statement
