# Supplementary material for: Seroprevalence of Antibodies to SARS-CoV-2 in Rural Households in Eastern Uganda, 2020-2022
Source: JAMA Netw Open. 2023 Feb 15;6(2):e2255978. doi: 10.1001/jamanetworkopen.2022.55978 (PMC9932849; doi:10.1001/jamanetworkopen.2022.55978)
Supplement: Supplement 1. — eAppendix. Supplemental Methods eTable 1. Number of Samples Seropositive for SARS-CoV-2 by Spike Protein MFI and Total Number of Samples Tested, by Serosurvey Round and Age Group eTable 2. Number of Participants With SARS-CoV-2 Seroconversion by Spike MFI and Total Number of Participants at Risk for Seroconversion, by Interval and Age Group eTable 3. Number of Participants With SARS-CoV-2 Boosting by Spike MFI and Total Number of Participants at Risk for Boosting, by Interval and Age Group eTable 4. Symptoms and Diagnoses Associated With SARS-CoV-2 Seroconversion eTable 5. Risk Factors for Antibody Boosting and Associations With Malaria eFigure 1. District-Level Weekly SARS-CoV-2 Case Counts in Eastern Uganda, Compared to in the Country Overall eFigure 2. Standard Dilution Series Used for Assay Normalization eFigure 3. Replicability of the Luminex Assay eFigure 4. The Age Distribution of This Study Population, Compared to the Age Distribution of the Negative Control Samples Used eFigure 5. Spike Protein Antibody Responses for SARS-CoV-2 Negative Control and Positive Control Samples eFigure 6. Longitudinal Antibody Kinetics for 11 Participants in the Cohort Study Who Had Confirmed SARS-CoV-2 Infections eFigure 7. Scatterplot of Agreement Between Measured MFIs and Inferred Antibody Concentrations eFigure 8. Antibody Responses to Additional SARS-CoV-2 Antigens Tested eFigure 9. Timing of SARS-CoV-2 Vaccination eFigure 10. SARS-CoV-2 Antibody Kinetics, Stratified by Age Group and by Serosurvey Round at Which an Individual’s Antibody Response Was First Positive eFigure 11. SARS-CoV-2 Antibody Boosting Between Rounds 3 and 4 in Vaccinated Individuals, Stratified by Vaccine Type eFigure 12. Antibody Boosting by Strata eFigure 13. Sensitivity Analyses for SARS-CoV-2 Antibody Boosting Between Rounds 3 and 4 Using Alternative Cutoffs eFigure 14. Sensitivity Analyses for SARS-CoV-2 Antibody Boosting Between Rounds 3 and 4 Using Nucleocapsid Protein Responses eFigure 15. Pairwi [file jamanetwopen-e2255978-s001.pdf]

Supplemental Online Content

Briggs J, Takahashi S, Nayebare P, et al. Seroprevalence of antibodies to SARS-CoV-2 in rural households in eastern Uganda, 2020-2022. *JAMA Netw Open*. 2023;6(2):e2255978. doi:10.1001/jamanetworkopen.2022.55978

eAppendix. Supplemental Methods
eTable 1. Number of Samples Seropositive for SARS-CoV-2 by Spike Protein MFI and Total Number of Samples Tested, by Serosurvey Round and Age Group
eTable 2. Number of Participants With SARS-CoV-2 Seroconversion by Spike MFI and Total Number of Participants at Risk for Seroconversion, by Interval and Age Group
eTable 3. Number of Participants With SARS-CoV-2 Boosting by Spike MFI and Total Number of Participants at Risk for Boosting, by Interval and Age Group
eTable 4. Symptoms and Diagnoses Associated With SARS-CoV-2 Seroconversion
eTable 5. Risk Factors for Antibody Boosting and Associations With Malaria
eFigure 1. District-Level Weekly SARS-CoV-2 Case Counts in Eastern Uganda, Compared to in the Country Overall
eFigure 2. Standard Dilution Series Used for Assay Normalization
eFigure 3. Replicability of the Luminex Assay
eFigure 4. The Age Distribution of This Study Population, Compared to the Age Distribution of the Negative Control Samples Used
eFigure 5. Spike Protein Antibody Responses for SARS-CoV-2 Negative Control and Positive Control Samples
eFigure 6. Longitudinal Antibody Kinetics for 11 Participants in the Cohort Study Who Had Confirmed SARS-CoV-2 Infections
eFigure 7. Scatterplot of Agreement Between Measured MFIs and Inferred Antibody Concentrations
eFigure 8. Antibody Responses to Additional SARS-CoV-2 Antigens Tested
eFigure 9. Timing of SARS-CoV-2 Vaccination
eFigure 10. SARS-CoV-2 Antibody Kinetics, Stratified by Age Group and by Serosurvey Round at Which an Individual’s Antibody Response Was First Positive
eFigure 11. SARS-CoV-2 Antibody Boosting Between Rounds 3 and 4 in Vaccinated Individuals, Stratified by Vaccine Type
eFigure 12. Antibody Boosting by Strata
eFigure 13. Sensitivity Analyses for SARS-CoV-2 Antibody Boosting Between Rounds 3 and 4 Using Alternative Cutoffs
eFigure 14. Sensitivity Analyses for SARS-CoV-2 Antibody Boosting Between Rounds 3 and 4 Using Nucleocapsid Protein Responses
eFigure 15. Pairwise Odds Ratios for Clustering of SARS-CoV-2 Seroconversions Within Households, by Time Period and by Age Category

This supplemental material has been provided by the authors to give readers additional information about their work.

© 2023 Briggs J et al. *JAMA Network Open.*

eAppendix. Supplemental Methods

PRISM Border Cohort study enrollment: All households in the parishes of Osukuru, Kayoro, and Buteba parishes in eastern Uganda were enumerated to generate a sampling frame to recruit households into the PRISM Border Cohort study. In August 2020, enumerated households were randomly selected and screened for eligibility to participate. Inclusion criteria for a household to participate included having at least two members aged 5 years or younger. All permanent members of an enrolled household who met eligibility criteria were screened for study enrollment. The cohort was dynamic, so any permanent members that joined an enrolled household were also screened for enrollment.

Covalent antigen coupling to magnetic microsphere beads: Antigens were covalently coupled to MagPlex carboxylated magnetic microspheres (Luminex Corp, Austin, USA). 12.5×10^6 beads for each bead region were suspended by vortexing at medium speed for 2 minutes. The beads were transferred from the stock bottle into a 1.5ml micro-centrifuge tube with low protein binding surface (Eppendorf, UK) to minimize bead loss and centrifuged at 16,000g for 5 minutes. The bead pellet was pulled on the tube side by placing into a magnetic rack for 2 minutes before pipetting off the supernatant. The beads were washed twice by suspension in 1000µl distilled water, vortexed for 30 seconds and centrifuged at 16,000g for 5 minutes. Supernatant was removed after settling the beads on a magnetic rack for 2 minutes. Beads were suspended in 800µl monobasic Sodium Phosphate (NaH_2PO_4, pH 6.2 activation buffer). To activate the carboxyl surface of the beads, 100µl of 50mg/ml Hydroxysulfosuccinimide (Sulfo-NHS) (Thermo Fisher scientific, UK) was added, vortexed briefly and immediately followed by addition of 100µl of 50mg/ml 1-ethyl-3-(3-dimethylaminopropyl) carbodiimide hydrochloride (EDC) (Thermo Fisher scientific, UK). The beads were incubated for 20 minutes at room temperature in the dark, wrapped in aluminum foil on a plate shaker. Beads were pelleted by centrifuging at 16,000g for 5 minutes, placed tubes in a magnetic holder for 2 minutes, the supernatant carefully removed, and washed 3 times with 1000µl PBS. Previously determined amounts of antigen (38.5µl/ml spike, 42ug/ml RBD, and 29.8ug/ml nucleocapsid proteins) were added to each bead region in PBS to make a final volume of 1000µl. The beads/antigen suspension was incubated for 2 hours on a rotating shaker in the dark, covered in aluminum foil. Coupled beads were pelleted by centrifugation at 16,000g for 5 minutes and washed of excess antigen 3 times with 1000µl of phosphate buffered saline 0.05% tween20 (PBS-TBN). The antigen coupled beads were suspended in 1000µl storage buffer (PBS-TBN-Sodium Azide) and stored at 4°C.

Assay to measure total IgG: Total IgG responses to spike, RBD, and nucleocapsid proteins were assayed in plasma at 1:400 dilution using the multiplex bead array using the Luminex Magpix machine (Luminex Corp, Austin, Texas). 10ul of each of the three coupled bead regions was added to 5ml PBS-TBN. 50µl of the pooled bead suspension was added to each well, washed and incubated with 50µl of 1:400 test plasma or control sample for 1.5 hours at room temperature. The plates were washed and 50ul of 1/250 goat anti-human IgG rPE labeled antibody (Jackson Immuno Research Laboratories) was added and incubated for 1.5 hours on a shaking platform. The plates were washed, 1xPBS added and read on a MagPix machine.The blank well MFI was deducted from each well to determine the net MFI. We required a minimum of 50 beads per analyte for a measurement to be included in this analysis. Cutoff for seropositivity was established as the highest value among the negative controls; this was determined to be 516 for background-subtracted spike median MFI, and 0.024 for relative spike antibody concentration.

Estimating seroprevalence and attack rate using a Bayesian measurement model: The test performance characteristics of a single assay (i.e., sensitivity (Se) and specificity (Sp)) can be

© 2023 Briggs J et al. *JAMA Network Open.*

determined from a 2 x 2 table of positive/negative control samples and their binary classification on that assay using a binomial model as follows:

$$y\_pos \sim \text{Binomial}(N\_pos, Se) \quad \text{(Equation 1)}$$

$$y\_neg \sim \text{Binomial}(N\_neg, Sp) \quad \text{(Equation 2)}$$

In Equation 1, N_pos represents the total number of positive control samples tested and y_pos represents the number of positive control samples that tested positive. In Equation 2, N_neg represents the total number of negative control samples tested and y_neg represents the number of negative control samples that tested negative. For a given serosurvey where N total samples were tested and y samples tested positive, the adjusted seroprevalence p_adj can be estimated using the binomial model in Equation 3:

$$y \sim \text{Binomial}(N, p\_adj \cdot Se + (1 - p\_adj) \cdot (1 - Sp)) \quad \text{Equation 3)}$$

The same approach can be used to estimate the attack rate.

Testing for clustering of seroconversions within households using pairwise odds ratios: The pairwise odds ratio is the odds of seroconversion for an individual in a household if another individual from the same household seroconverted, relative to their odds of seroconversion if the individual did not seroconvert. Pairwise odds ratio values greater than 1 indicate clustering within households. We estimated within-age category and between-age category pairwise odds ratios by time interval, and assumed that the pairwise odds ratios were constant across all households. The probabilities and odds were estimated in a multinomial Bayesian framework with a 4-simplex distribution parameter representing the possible serostatus permutations in a pair of individuals.

© 2023 Briggs J et al. *JAMA Network Open.*

eTable 1: Number of Samples Seropositive for SARS-CoV-2 by Spike Protein MFI and Total Number of Samples Tested, by Serosurvey Round and Age Group

Serosurvey round	Age group	Seropositive	Total
1	< 5 years	14	89
1	5-15 years	15	69
1	16 years or older	25	87
1	All ages	54	245
2	< 5 years	35	134
2	5-15 years	40	126
2	16 years or older	82	154
2	All ages	157	414
3	< 5 years	74	153
3	5-15 years	68	129
3	16 years or older	118	152
3	16 years or older, unvaccinated at Round 3	115	149
3	All ages	260	434
4	< 5 years	115	139
4	5-15 years, total	107	114
4	5-15 years, unvaccinated at Round 4	107	113
4	16 years or older, total	133	137
4	16 years or older, unvaccinated at Round 4	30	32
4	All ages	355	390

© 2023 Briggs J et al. *JAMA Network Open.*

eTable 2: Number of Participants With SARS-CoV-2 Seroconversion by Spike MFI and Total Number of Participants at Risk for Seroconversion, by Interval and Age Group
Individuals are removed from the risk set if they had received SARS-CoV-2 vaccination before or during the interval.

Interval	Age group	Seroconverted during interval	Total at risk for seroconversion during interval
Round 1 to Round 2	< 5 years	13	68
Round 1 to Round 2	5-15 years	11	52
Round 1 to Round 2	16 years or older	30	62
Round 1 to Round 2	All ages	54	182
Round 2 to Round 3	< 5 years	32	97
Round 2 to Round 3	5-15 years	28	86
Round 2 to Round 3	16 years or older	37	69
Round 2 to Round 3	All ages	97	252
Round 3 to Round 4	< 5 years	49	72
Round 3 to Round 4	5-15 years	50	55
Round 3 to Round 4	16 years or older	6	7
Round 3 to Round 4	All ages	105	134

© 2023 Briggs J et al. *JAMA Network Open.*

eTable 3: Number of Participants With SARS-CoV-2 Boosting by Spike MFI and Total Number of Participants at Risk for Boosting, by Interval and Age Group. Only participants who were seropositive at Round 3 were considered at risk for boosting. Boosting was defined as a ≥ 4 fold increase in MFI between Round 3 and Round 4.

Age group	Vaccination status	Boosted during interval	Total at risk for boosting during interval
< 5 years	Unvaccinated at Round 4	15	65
5-15 years	Unvaccinated at Round 4	23	58
16 years or older	Unvaccinated at Round 4	17	25
16 years or older	Vaccinated between Round 3 and Round 4	68	81
16 years or older	Vaccinated between Round 2 and Round 3	2	3
All ages	--	125	232

© 2023 Briggs J et al. *JAMA Network Open.*

eTable 4: Symptoms and Diagnoses Associated With SARS-CoV-2 Seroconversion.
Diarrhea, fever, muscle aches, and other diagnostic categories assessed were not associated with seroconversion.

Characteristic	OR[1]	95% CI[1]	p-value
**Cough**	1.44	1.02, 2.02	**0.037**
**Headache**	2.04	1.43, 2.92	**<0.001**
**Fatigue**	1.82	1.04, 3.21	**0.037**
**Total number sick visits**	1.17	1.07, 1.29	**<0.001**
**Any URTI diagnosis**	1.86	1.21, 2.87	**0.005**
**Total number URTI diagnoses**	1.67	1.18, 2.35	**0.003**

[1]OR = Odds Ratio, CI = Confidence Interval

© 2023 Briggs J et al. *JAMA Network Open.*

eTable 5: Risk Factors for Antibody Boosting and Associations With Malaria.

Characteristic	OR[1]	95% CI[1]	p-value
**Age Category**			
< 5 years	—	—	
5-15 years	2.19	1.01, 4.86	**0.049**
**Gender**			
Female	—	—	
Male	0.93	0.43, 2.01	0.86
**Wealth Tertile**			
Lowest	—	—	
Middle	0.76	0.28, 2.17	0.61
Highest	0.42	0.14, 1.24	0.11
**Housing Type**			
Modern	—	—	
Traditional	0.46	0.21, 1.01	0.053
**Sanitation**			
Uncovered pit latrine or no facility	—	—	
VIP or covered pit latrine	1.17	0.50, 2.67	0.71
**Number of people in household**	1.26	0.92, 1.77	0.16
**Malaria incidence**	0.95	0.79, 1.12	0.60
**Any asymptomatic parasitemia**	1.60	0.74, 3.49	0.23
**Number of episodes of asymptomatic parasitemia**	1.37	1.05, 1.81	**0.023**

[1]OR = Odds Ratio, CI = Confidence Interval

© 2023 Briggs J et al. *JAMA Network Open.*

eFigure 1: District-Level Weekly SARS-CoV-2 Case Counts in Eastern Uganda, Compared to in the Country Overall. In blue and on the left y-axis: aggregated weekly SARS-CoV-2 positive results from the 20 districts in eastern Uganda highlighted on the inset map (Budaka, Bugiri, Bugweri, Buikwe, Bukwo, Bulambuli, Busia, Butaleja, Butebo, Iganga, Jinja, Kaliro, Kibuku, Luuka, Mayuge, Mbale, Namisindwa, Namutumba, Pallisa, and Sironko). On the right y-axis: reported daily new COVID-19 cases in all of Uganda, replicated from Figure 1A.

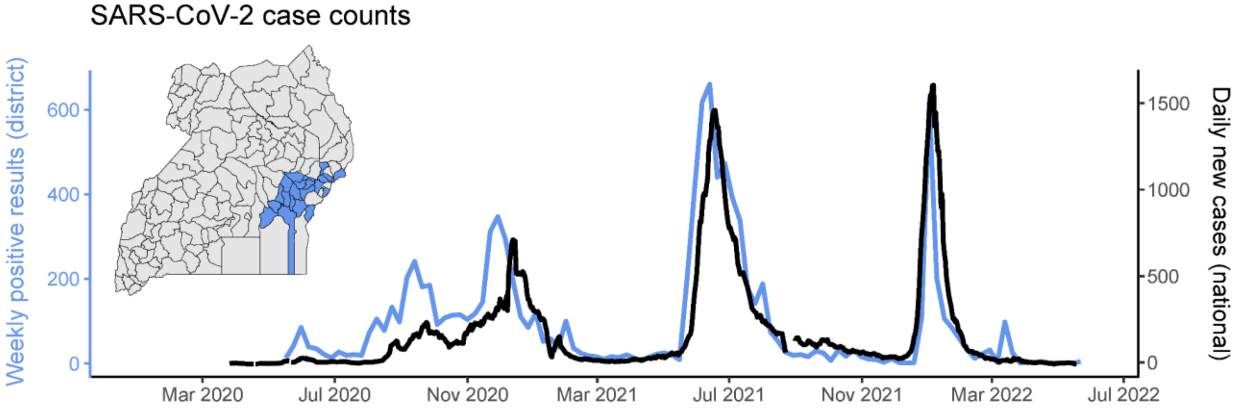

© 2023 Briggs J et al. *JAMA Network Open.*

eFigure 2: Standard Dilution Series Used for Assay Normalization. The x-axis represents the log antibody concentration and the y-axis represents the median MFI value of the spike protein response. The black points indicate the serial dilution (inverse of concentration) of the standards. Each line represents a plate, with 32 plates tested in total. The background response (average of blank wells) for each plate is shown in blue.

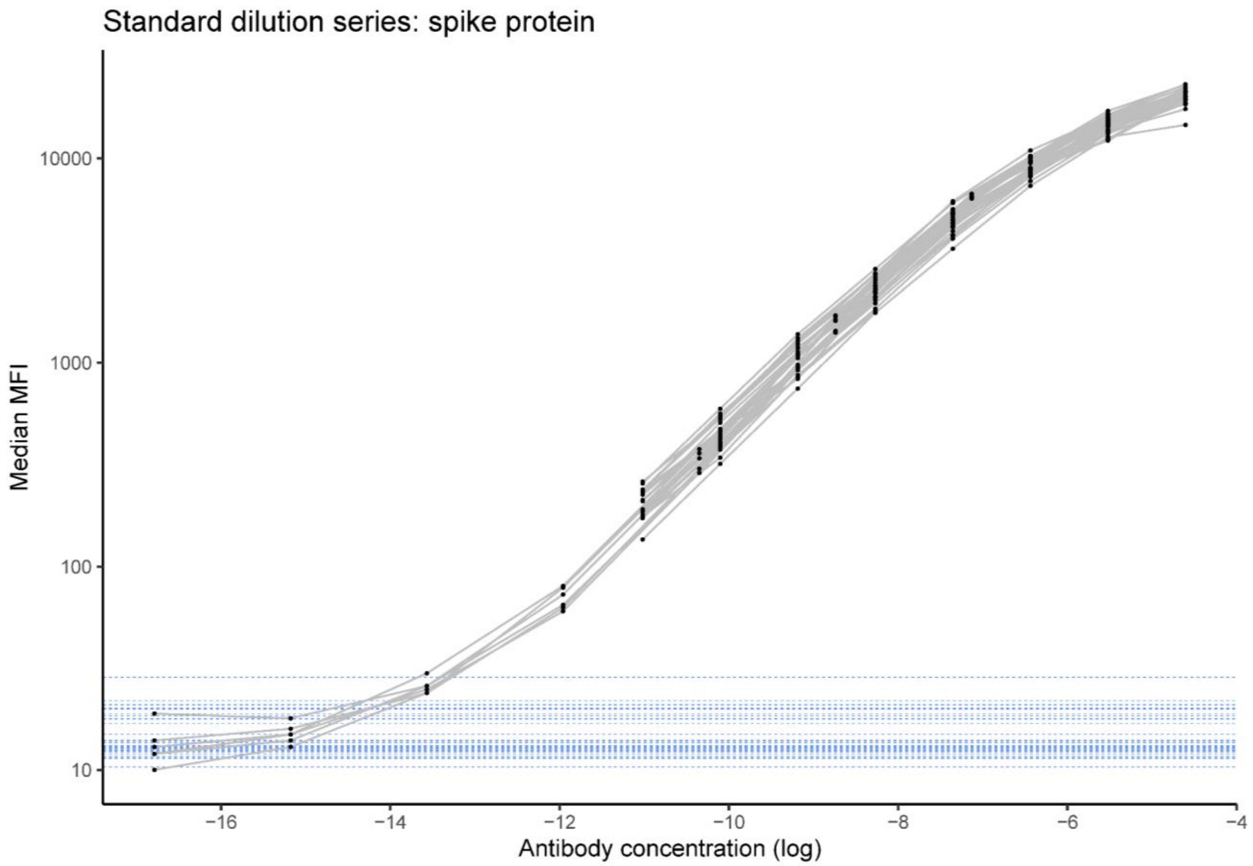

© 2023 Briggs J et al. *JAMA Network Open.*

eFigure 3: Replicability of the Luminex Assay. Scatterplots of spike protein **(A)** MFIs and **(B)** antibody concentrations for 50 samples tested in replicate. The cutoff for seropositivity for each metric, determined based on the highest negative control, is shown in blue. On Panel B, samples that were below the limit of quantitation for antibody concentrations (i.e., below the lowest standard dilution on the plate) are shown in red.

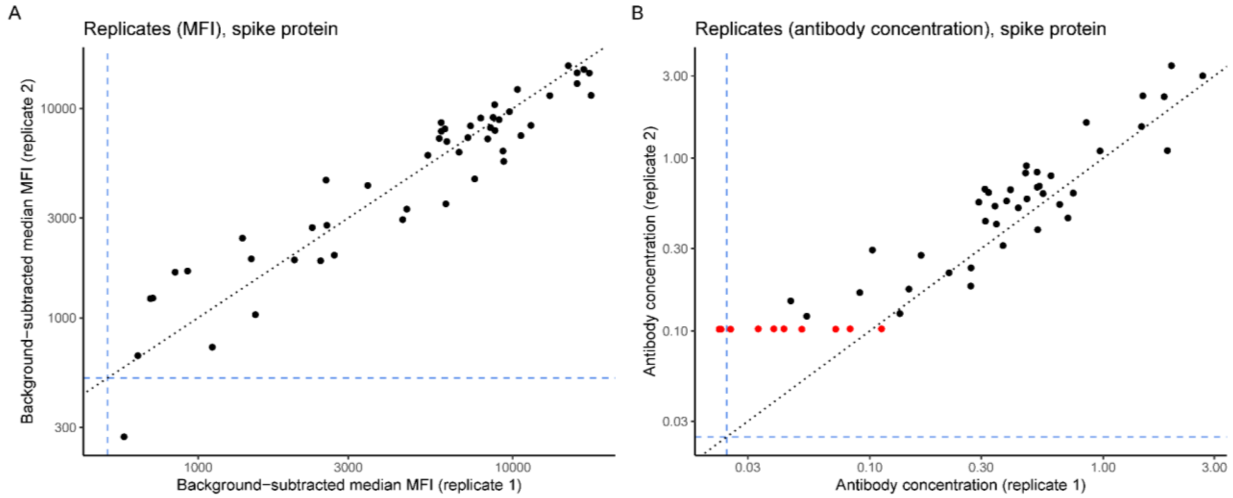

© 2023 Briggs J et al. *JAMA Network Open.*

eFigure 4: The Age Distribution of This Study Population, Compared to the Age Distribution of the Negative Control Samples Used.

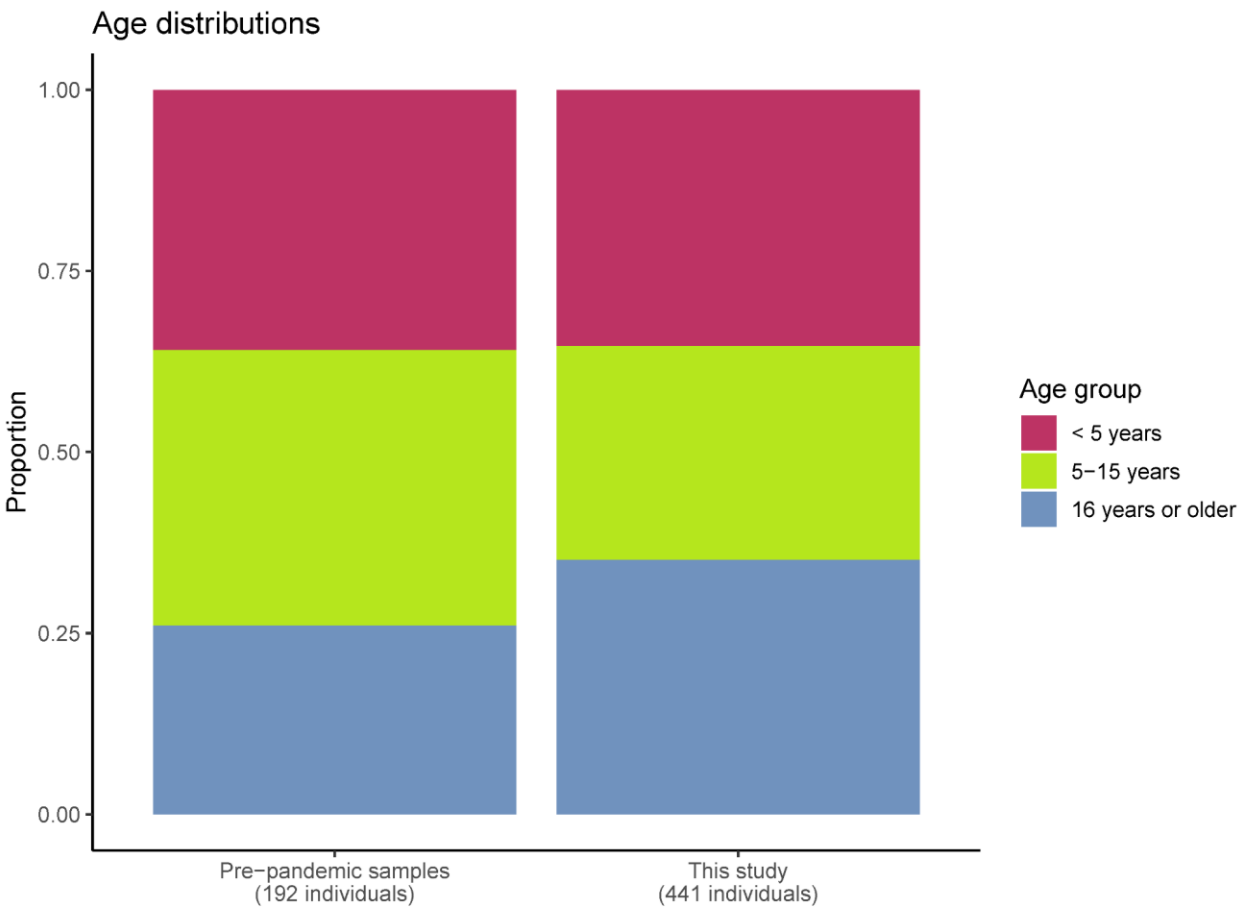

© 2023 Briggs J et al. *JAMA Network Open.*

eFigure 5: Spike Protein Antibody Responses for SARS-CoV-2 Negative Control and Positive Control Samples. The cutoff for seropositivity is shown in the blue dashed line (background-subtracted median MFI = 516).

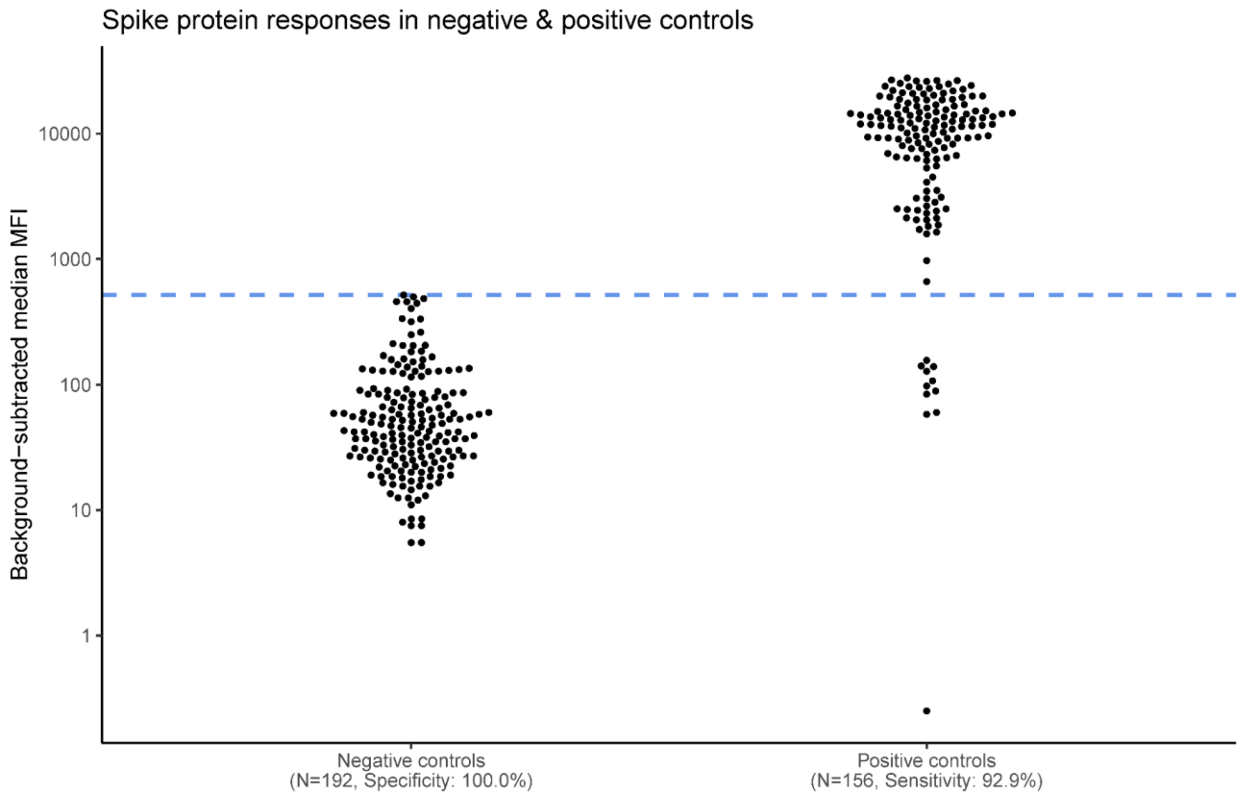

© 2023 Briggs J et al. *JAMA Network Open.*

eFigure 6: Longitudinal Antibody Kinetics for 11 Participants in the Cohort Study Who Had Confirmed SARS-CoV-2 Infections. The x-axis represents days since infection and the y-axis represents the spike protein antibody response. The 11 infections occurred between February and July 2021. Samples from Round 4 of the serosurvey are omitted from this visualization to preclude boosting effects. The cutoff for seropositivity is shown in the blue dashed line (background-subtracted median MFI = 516).

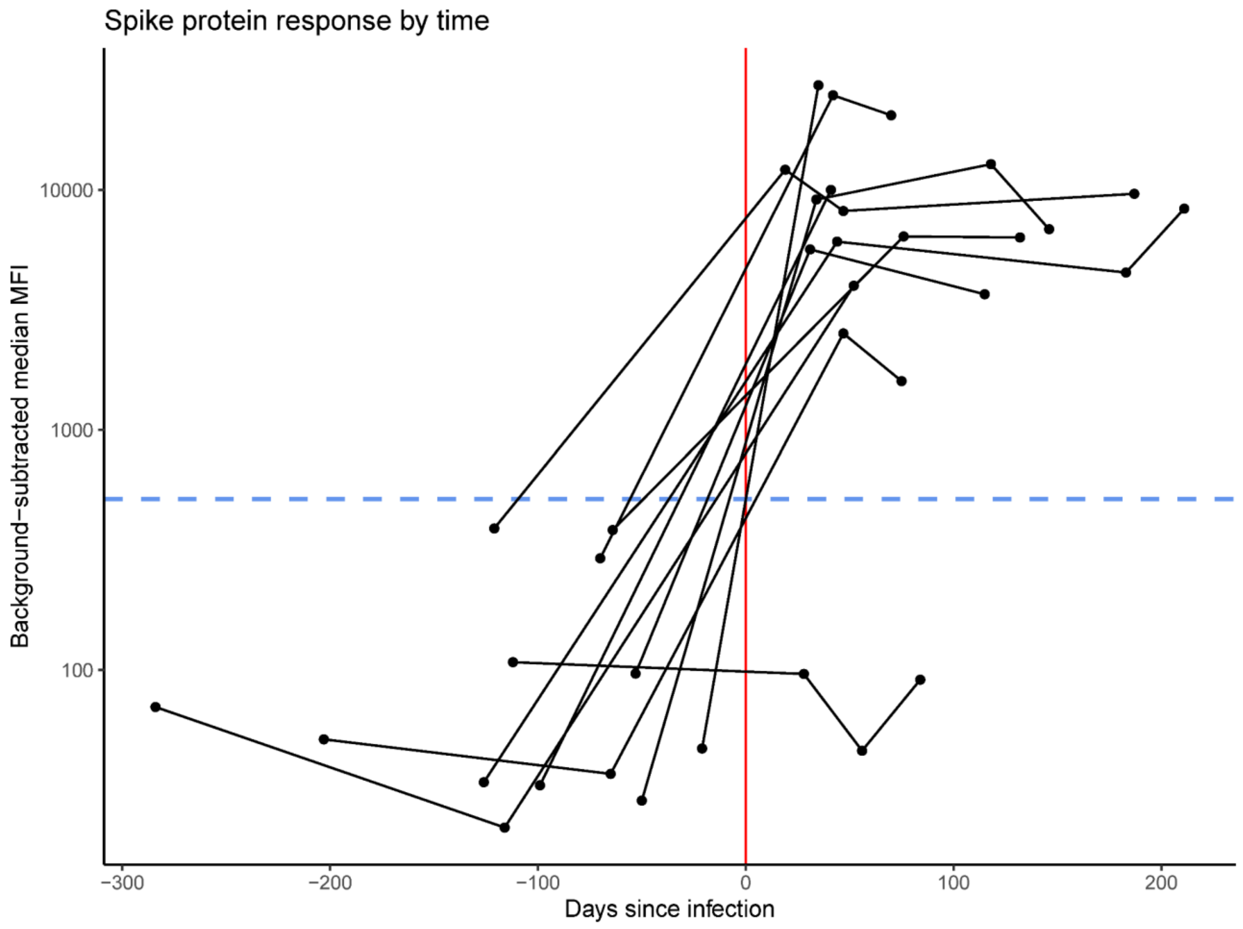

© 2023 Briggs J et al. *JAMA Network Open.*

eFigure 7: Scatterplot of Agreement Between Measured MFIs and Inferred Antibody Concentrations. Each point represents one of the 1,483 serosurvey samples tested at the primary concentration (1:400). Only 18 of 1,483 (1.2%) binary seropositivity results were not in agreement between MFIs and antibody concentrations, all of which were called positive using MFIs and negative using concentrations. The cutoff for seropositivity for each metric, determined based on the highest negative control, is shown in blue.

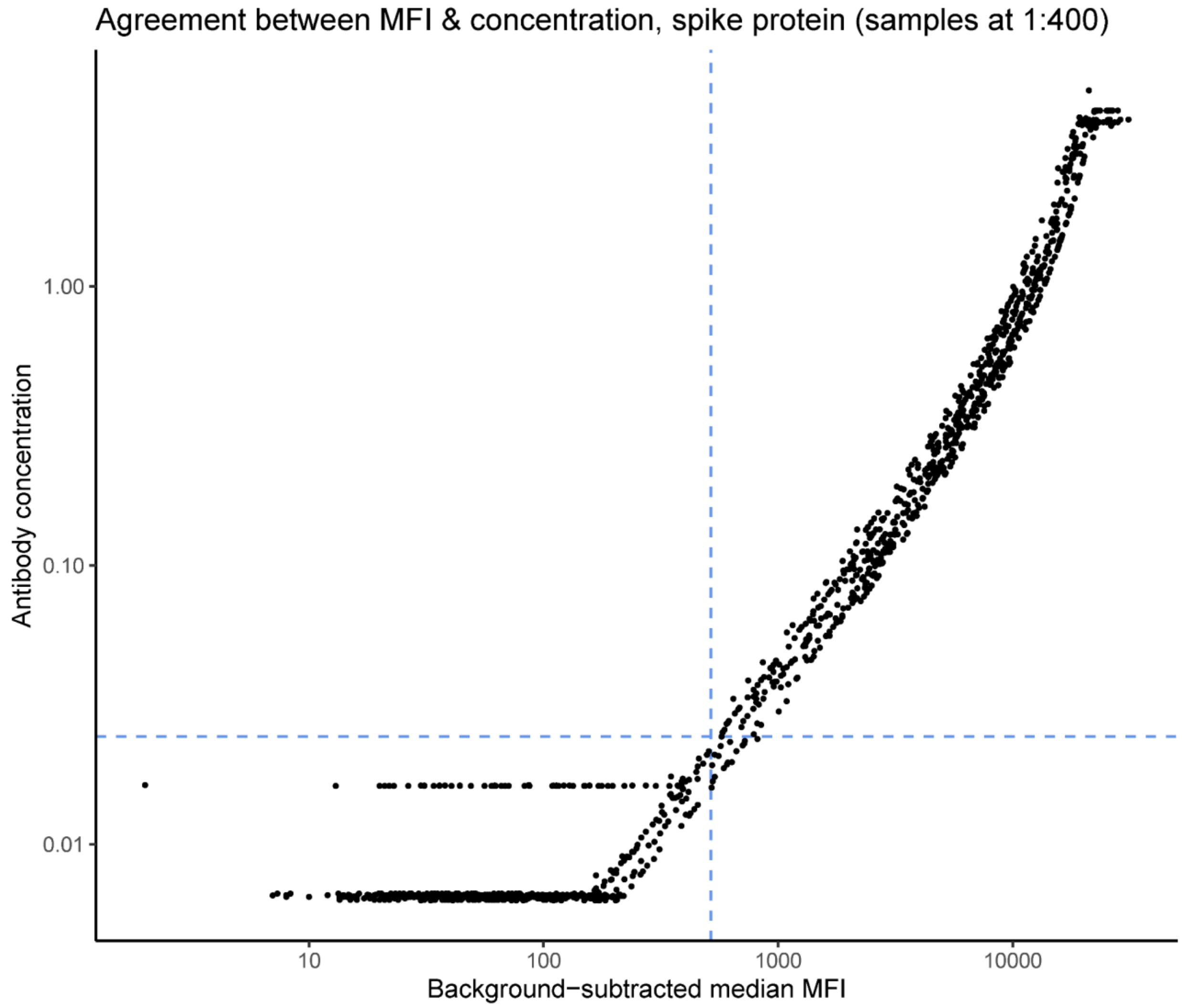

© 2023 Briggs J et al. *JAMA Network Open.*

eFigure 8: Antibody Responses to Additional SARS-CoV-2 Antigens Tested. Analogous to Figure 1B, here showing antibody responses to the **(A)** receptor-binding domain of the spike protein (RBD) and **(B)** nucleocapsid protein.

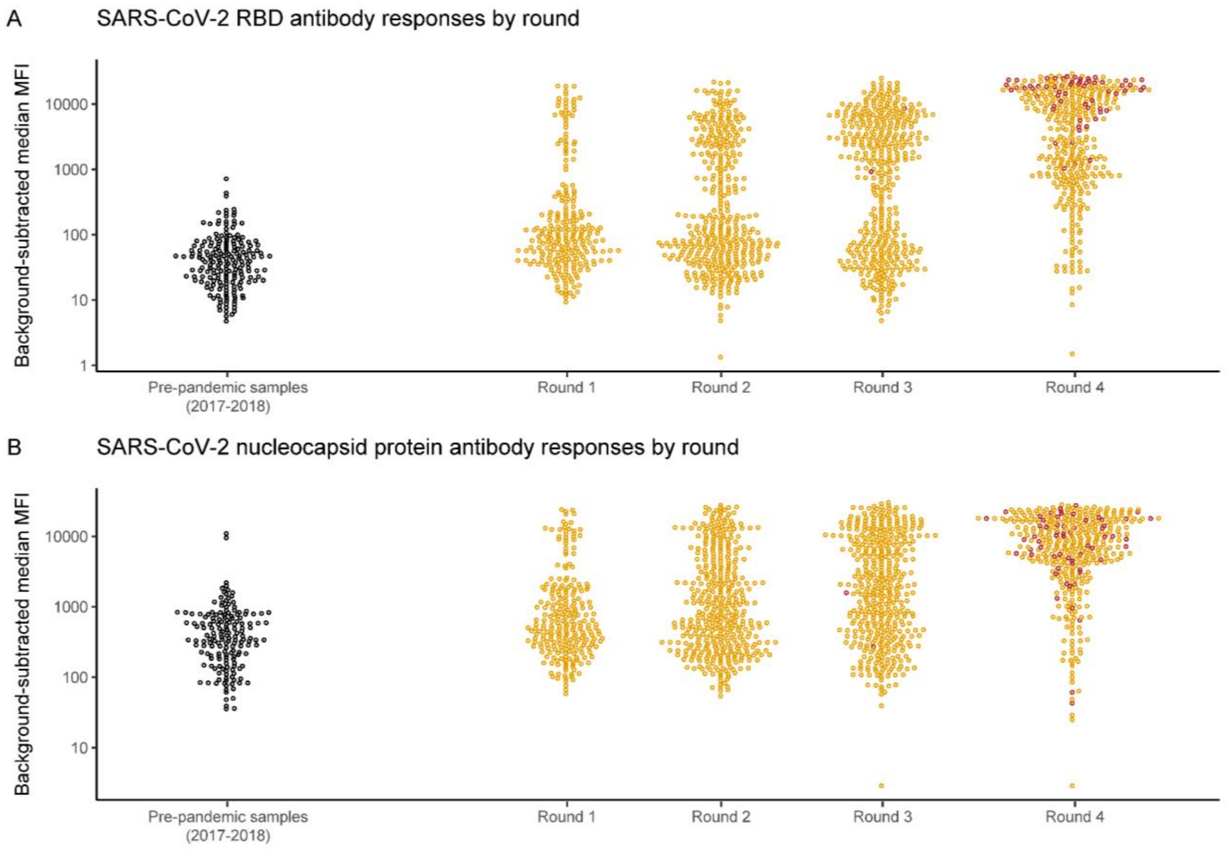

© 2023 Briggs J et al. *JAMA Network Open.*

eFigure 9: Timing of SARS-CoV-2 Vaccination. Days between **(A)** Round 3 and **(B)** Round 4 of the serosurvey and vaccination date. The times are stratified by whether vaccination was received within the 3 weeks prior to sample collection (pink), or 3 or more weeks prior to sample collection (turquoise). Negative values of the x-axis indicate vaccination that occurred before sample collection (i.e., panel A indicates that all but 3 participants who received vaccination were vaccinated after their Round 3 serosurvey sample). **(C)** Dates of SARS-CoV-2 vaccination among study participants. Vaccines received included 83 Janssen/Johnson & Johnson, 12 AstraZeneca, 9 Moderna, and 1 Sinovac.

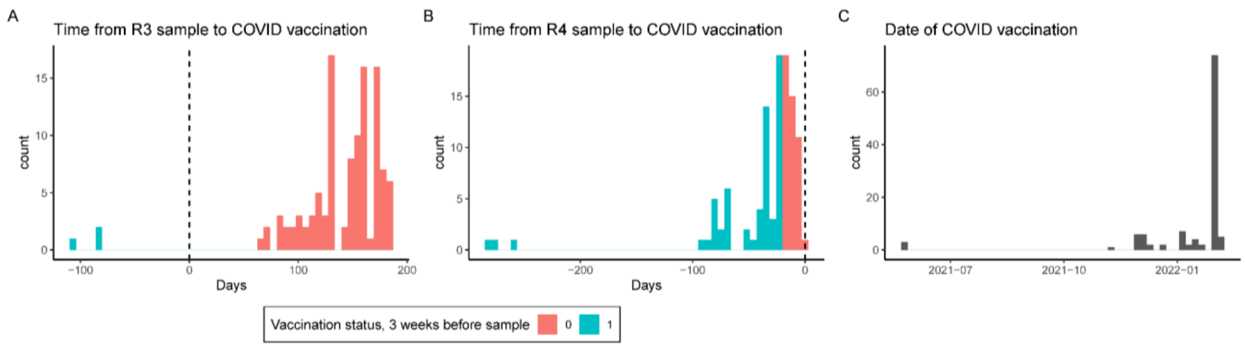

© 2023 Briggs J et al. *JAMA Network Open.*

eFigure 10: SARS-CoV-2 Antibody Kinetics, Stratified by Age Group and by Serosurvey Round at Which an Individual's Antibody Response Was First Positive. The kinetics for a single individual are shown by a line. The x-axis shows calendar time and the y-axis shows the background-subtracted median MFI of the spike protein antibody response. The bottom row indicates individuals whose antibody response was negative across the time period of this study. The value in the lower left of each panel indicates the number of individuals represented in that panel. The black circles indicate participants who had received SARS-CoV-2 vaccination by the 3 weeks prior to the serosurvey sample collection.

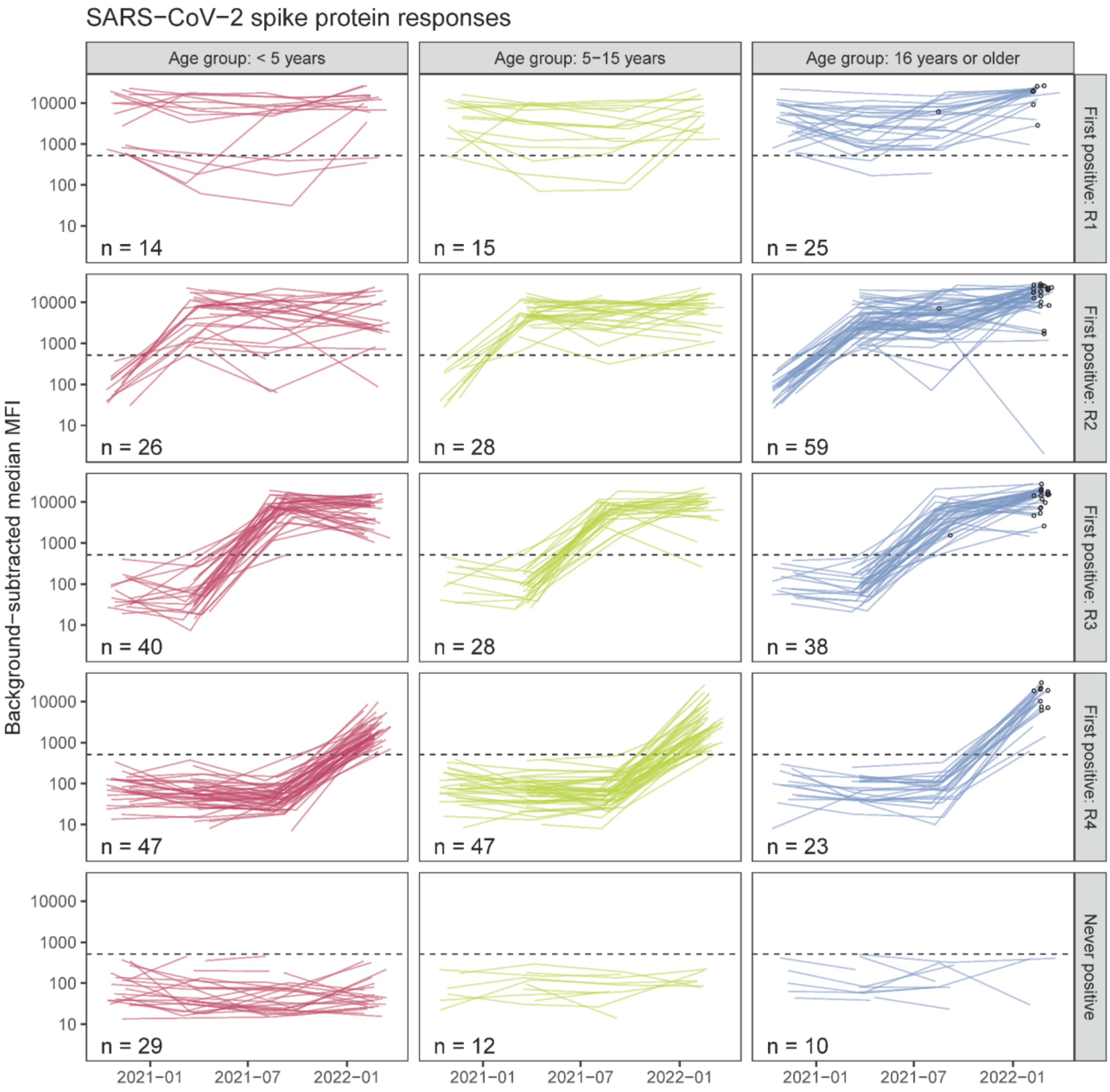

© 2023 Briggs J et al. *JAMA Network Open.*

eFigure 11: SARS-CoV-2 Antibody Boosting Between Rounds 3 and 4 in Vaccinated Individuals, Stratified by Vaccine Type. Analogous to Figure 3, boosting was defined as a ≥ 4 fold increase (orange line). The Round 3 antibody response is shown on the x-axis, and the fold change between the Round 4 and Round 3 antibody response is shown on the y-axis. This figure is limited to participants who were vaccinated at Round 4, and were separated by tertiles of Round 3 response (the second tertile is shown in the gray shaded rectangle). The proportion of individuals within each tertile that demonstrated antibody boosting is shown in orange text at the top. The colors of the points represent the manufacturer of the vaccine received.

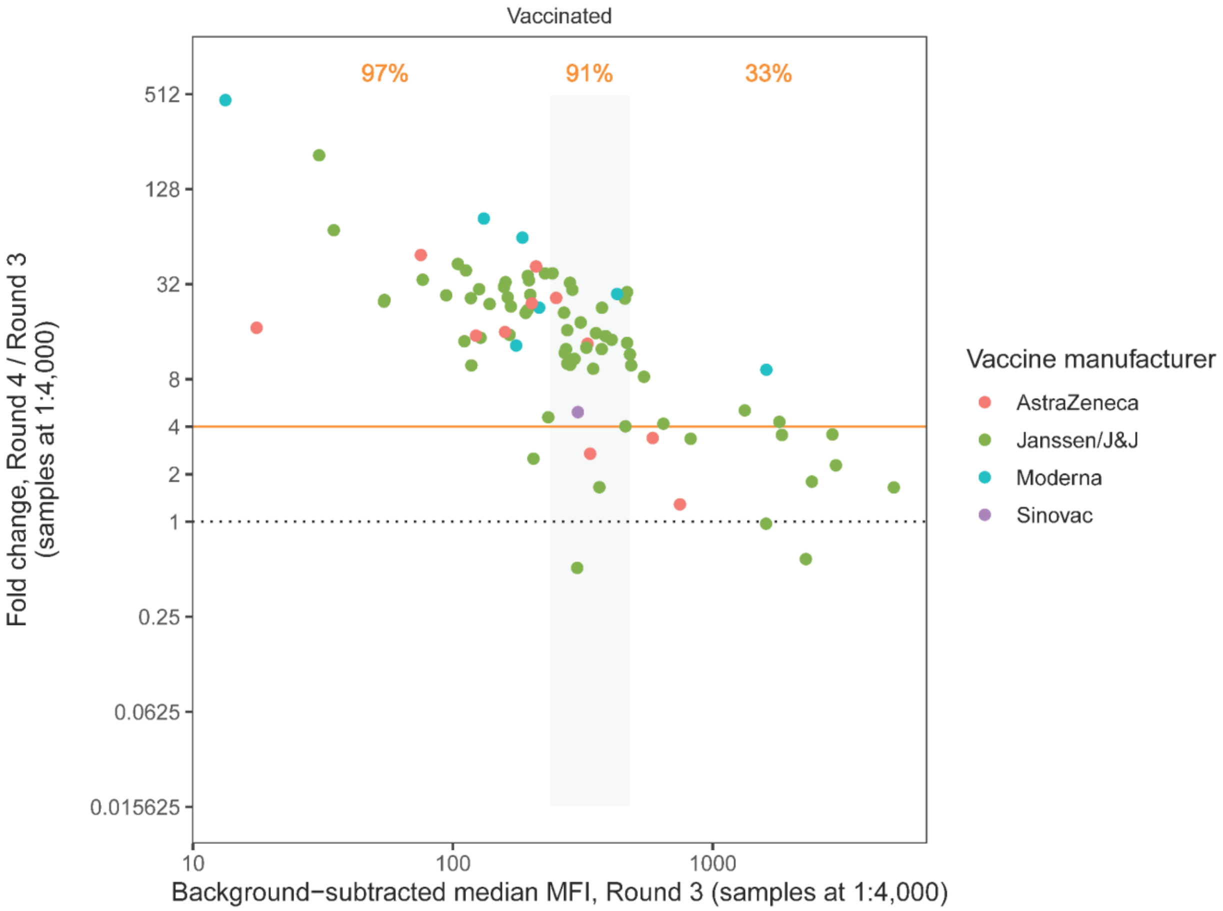

© 2023 Briggs J et al. *JAMA Network Open.*

eFigure 12: Antibody Boosting by Strata. (A) Probability of boosting among unvaccinated individuals only. The colors represent age group-specific estimates. The black values represent the crude estimates in the cohort. The gray values represent estimates weighted by the local age distribution using 2014 census data from the three parishes in Uganda in which study participants reside. **(B)** Relative risk of boosting by vaccination status at Round 4 and by baseline (Round 3) antibody tertile.

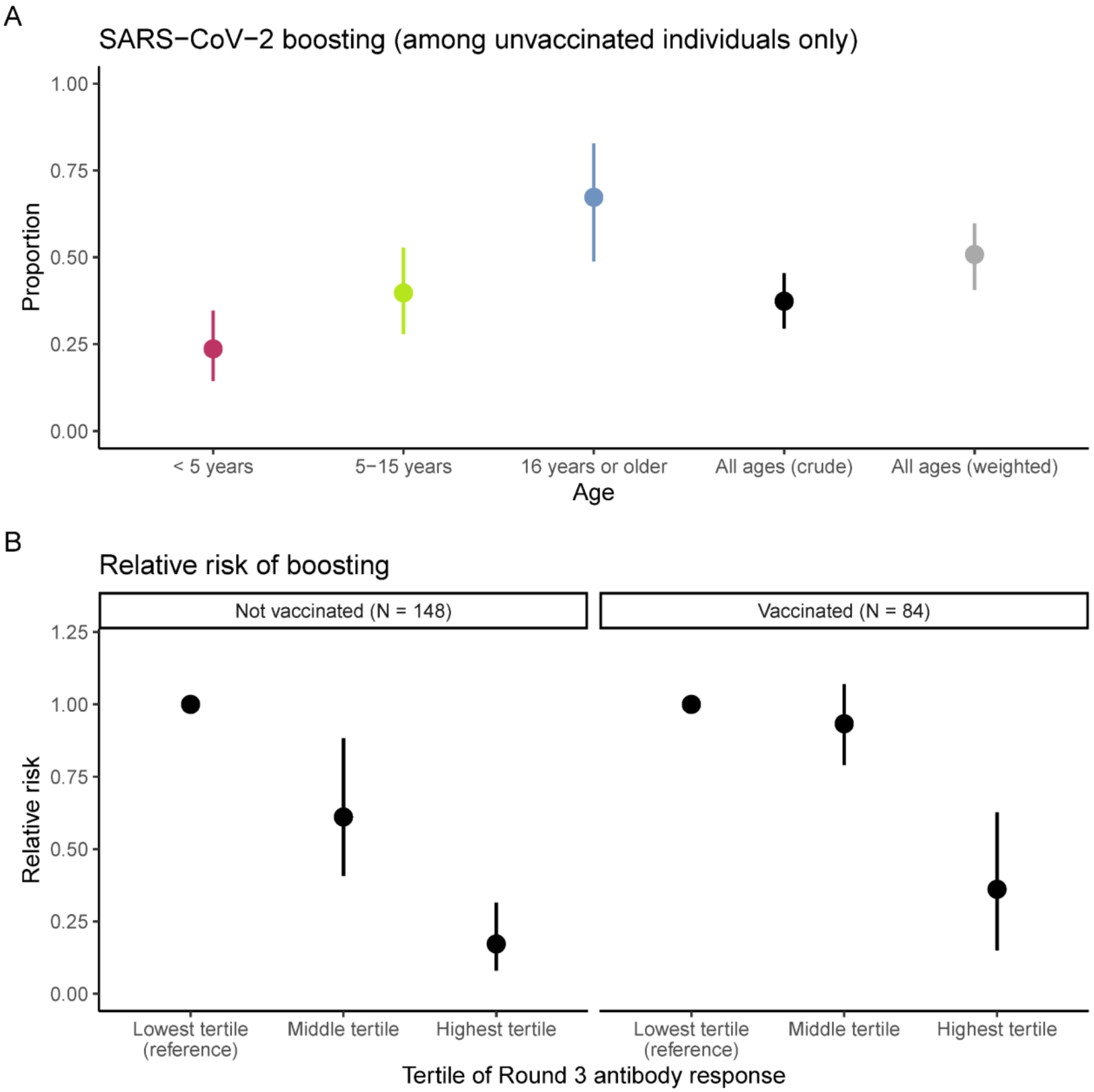

© 2023 Briggs J et al. *JAMA Network Open.*

eFigure 13: Sensitivity Analyses for SARS-CoV-2 Antibody Boosting Between Rounds 3 and 4 Using Alternative Cutoffs. Analogous to Figure 3, here showing different cutoffs of boosting: ≥ 2 fold increase in MFI (gold), ≥ 4 fold increase in MFI (orange), and ≥ 8 fold increase in MFI (purple) for spike protein. The Round 3 antibody response is shown on the x-axis, and the fold change between the Round 4 and Round 3 antibody response is shown on the y-axis. Participants were separated by vaccination status at Round 4 (panels) and by tertiles of Round 3 response (the second tertile is shown in the gray shaded rectangle). The proportion of individuals within each tertile that demonstrated antibody boosting is shown in gold, orange, and purple text at the top consistent with the respective colors of the lines representing cutoffs. The colors of the points represent age groups, and the shapes of the points represent binned time since SARS-CoV-2 vaccination at the Round 4 sample.

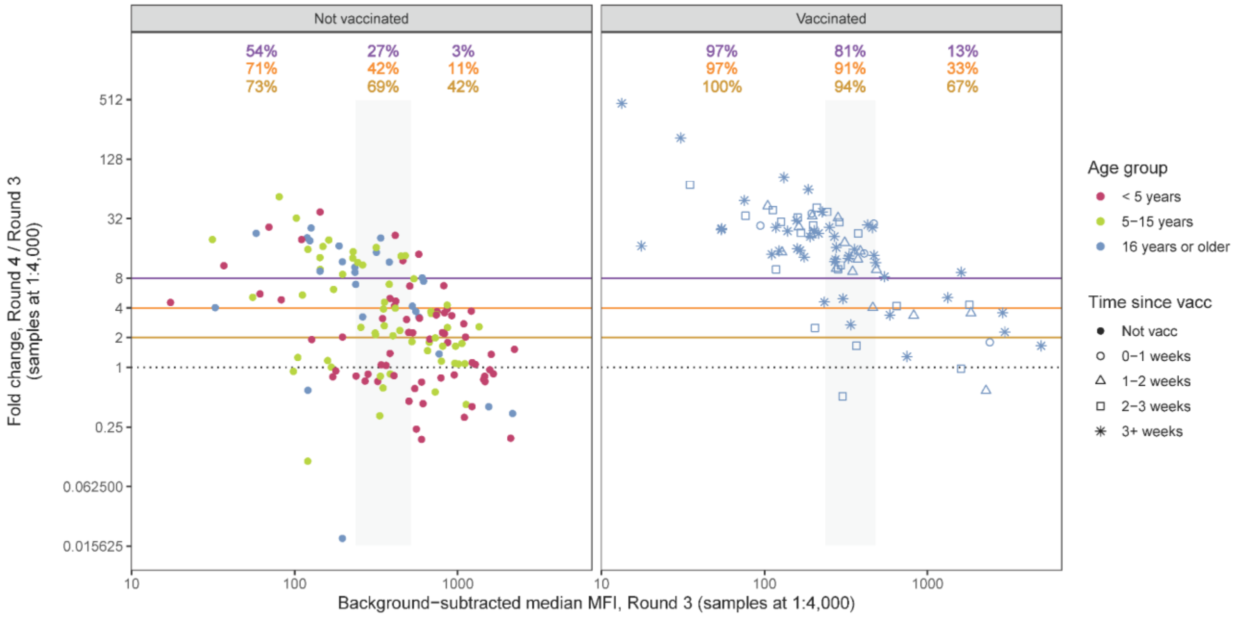

© 2023 Briggs J et al. *JAMA Network Open.*

eFigure 14: Sensitivity Analyses for SARS-CoV-2 Antibody Boosting Between Rounds 3 and 4 Using Nucleocapsid Protein Responses. (A) Fold change in spike (S, x-axis) or nucleocapsid (N, y-axis) between Round 3 and Round 4, for participants who were seropositive at Round 3 by spike. Note that vaccinated individuals on average had higher boosting to spike than nucleocapsid, consistent with inclusion of spike protein in the vaccine, but that some vaccinated individuals also had evidence of boosting with nucleocapsid, likely indicating concomitant re-infection. Four individuals with N responses that had less than the minimum of 50 beads per analyte were omitted. The dashed lines represent a 4 fold increase. **(B)** Frequency table of boosting of S and N responses by age group. Only participants who were seropositive at Round 3 by spike were considered at risk for boosting. Boosting was defined as a ≥ 4 fold increase in MFI for that protein between Round 3 and Round 4. **(C)** Age-weighted probability of boosting for each definition: boosting by S, boosting by N, boosting by S or N, boosting by S and N.

A

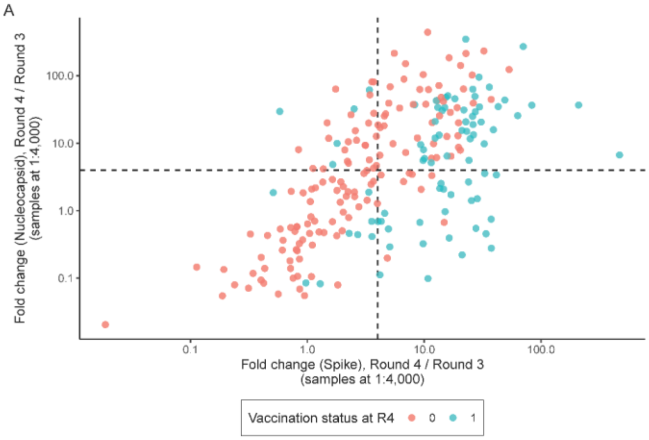

B

**< 5 years**	Boosted by N	Not boosted by N
Boosted by S	13	2
Not boosted by S	13	36
**5-15 years**	**Boosted by N**	**Not boosted by N**
Boosted by S	20	3
Not boosted by S	9	26
**16 years or older, unvaccinated at Round 4**	**Boosted by N**	**Not boosted by N**
Boosted by S	14	3
Not boosted by S	2	6

C

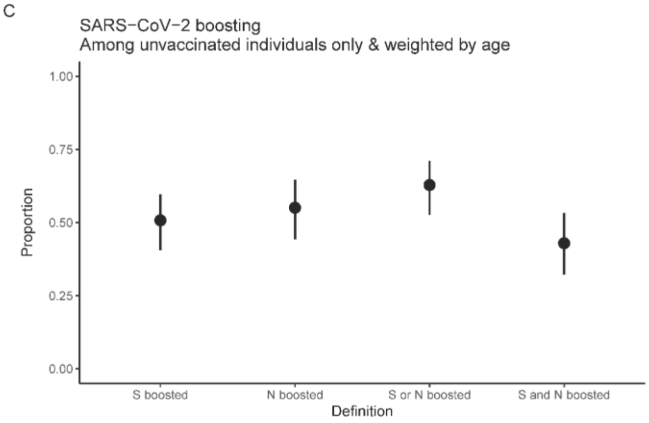

© 2023 Briggs J et al. *JAMA Network Open.*

eFigure 15: Pairwise Odds Ratios for Clustering of SARS-CoV-2 Seroconversions Within Households, by Time Period and by Age Category. The leftmost panel depicts results of pooling seroconversions across all intervals; the subsequent panels depict results stratified by interval. Individuals who had received SARS-CoV-2 vaccination by a serosurvey round are removed from the risk set for seroconversion. For each time period, we estimated within-age category and between-age category pairwise odds ratios for clustering within households (“Adult-Adult”, “Adult-Child”, “Child-Child”), as well as a single overall pairwise odds ratio for clustering within households (“Overall”). For this analysis, we defined children as individuals 15 years of age or younger, and adults are individuals 16 years of age or older. *A pairwise odds ratio for clustering within adults in households was not estimated for the interval between Round 3 and Round 4, as there were no households that had greater than 1 adult who was unvaccinated and susceptible. The dashed red line is for odds ratio = 1.

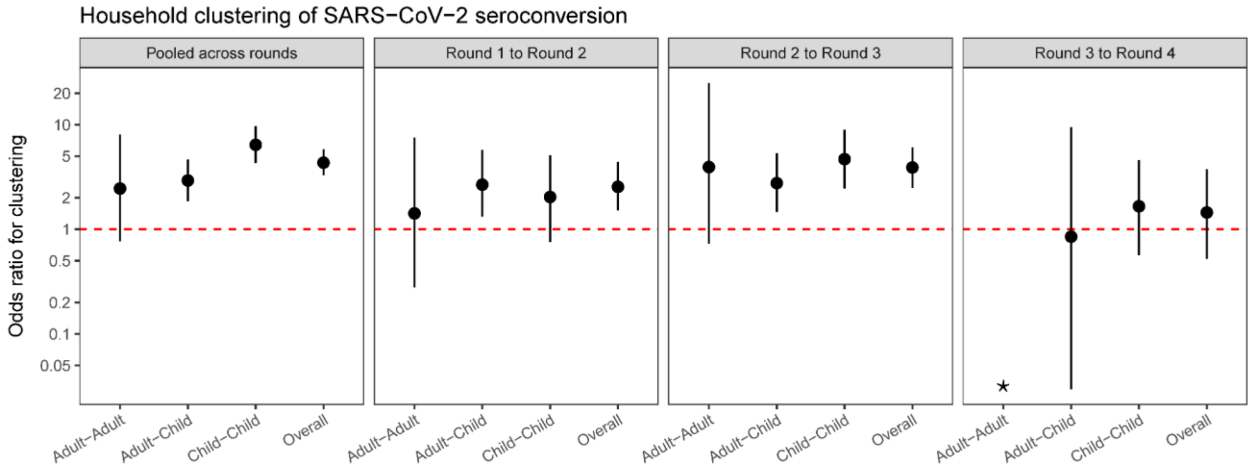

© 2023 Briggs J et al. *JAMA Network Open.*
