# Supplementary material for: Seroprevalence of Antibodies to SARS-CoV-2 in Rural Households in Eastern Uganda, 2020-2022
Source: JAMA Netw Open. 2023 Feb 15;6(2):e2255978. doi: 10.1001/jamanetworkopen.2022.55978 (PMC9932849; doi:10.1001/jamanetworkopen.2022.55978)
Supplement: Supplement 2. — Data Sharing Statement [file jamanetwopen-e2255978-s002.pdf]

Data Sharing Statement

Briggs. Seroprevalence of Antibodies to SARS-CoV-2 in Rural Households in Eastern Uganda, 2020-2022. *JAMA Netw Open*. Published February 15, 2023.
doi:10.1001/jamanetworkopen.2022.55978

Data
Data available: Yes
Data types: Deidentified participant data, Data dictionary
How to access data: Upon publication, data will be available here: https://github.com/EPPIcenter/PBC_Covid_serology. After the Prism Border Cohort study has been completed (which will occur after this publication date), all data pertaining to the cohort will be hosted using the ClinEpiDB platform: https://clinepidb.org/ce/app.
When available: With publication

Supporting Documents
Document types: None

Additional Information
Who can access the data: Data will be available to anyone through a public repository on Github.
Types of analyses: For any purpose.
Mechanisms of data availability: Data will be accessible without discussing with the investigators on this project.
